# Evaluating Potential Response-Modifying Factors for Associations between Ozone and Health Outcomes: A Weight-of-Evidence Approach

**DOI:** 10.1289/ehp.1307541

**Published:** 2014-06-13

**Authors:** Lisa C. Vinikoor-Imler, Elizabeth O. Owens, Jennifer L. Nichols, Mary Ross, James S. Brown, Jason D. Sacks

**Affiliations:** 1 National Center for Environmental Assessment (NCEA), U.S. Environmental Protection Agency (EPA), Research Triangle Park, North Carolina, USA; 2 Oak Ridge Institute for Science and Education, NCEA, Office of Research and Development, U.S. EPA, Research Triangle Park, North Carolina, USA

## Abstract

Background: Epidemiologic and experimental studies have reported a variety of health effects in response to ozone (O_3_) exposure, and some have indicated that certain populations may be at increased or decreased risk of O_3_-related health effects.

Objectives: We sought to identify potential response-modifying factors to determine whether specific groups of the population or life stages are at increased or decreased risk of O_3_-related health effects using a weight-of-evidence approach.

Methods: Epidemiologic, experimental, and exposure science studies of potential factors that may modify the relationship between O_3_ and health effects were identified in U.S. Environmental Protection Agency’s 2013 *Integrated Science Assessment for Ozone and Related Photochemical Oxidants*. Scientific evidence from studies that examined factors that may influence risk were integrated across disciplines to evaluate consistency, coherence, and biological plausibility of effects. The factors identified were then classified using a weight-of-evidence approach to conclude whether a specific factor modified the response of a population or life stage, resulting in an increased or decreased risk of O_3_-related health effects.

Discussion: We found “adequate” evidence that populations with certain genotypes, preexisting asthma, or reduced intake of certain nutrients, as well as different life stages or outdoor workers, are at increased risk of O_3_-related health effects. In addition, we identified other factors (i.e., sex, socioeconomic status, and obesity) for which there was “suggestive” evidence that they may increase the risk of O_3_-related health effects.

Conclusions: Using a weight-of-evidence approach, we identified a diverse group of factors that should be considered when characterizing the overall risk of health effects associated with exposures to ambient O_3_.

Citation: Vinikoor-Imler LC, Owens EO, Nichols JL, Ross M, Brown JS, Sacks JD. 2014. Evaluating potential response-modifying factors for associations between ozone and health outcomes: a weight-of-evidence approach. Environ Health Perspect 122:1166–1176; http://dx.doi.org/10.1289/ehp.1307541

## Introduction

As discussed in the Clean Air Act Amendments of 1990, the health-based, or primary, National Ambient Air Quality Standards (NAAQS) for the criteria air pollutants [U.S. Environmental Protection Agency (EPA) 2011b], which include ozone (O_3_), are intended to provide an adequate margin of safety that is requisite to protect public health from ambient air pollution, taking into consideration “measures which may be employed to … protect the health of sensitive or susceptible individuals or groups” (Clean Air Act Amendments 1990). Therefore, as part of the NAAQS process, it is important to thoroughly evaluate the available scientific evidence to accurately identify those populations or life stages at increased risk of an air pollutant–related health effect. The most recent review of the scientific evidence that supports the NAAQS for O_3_ included an evaluation of factors that may increase or decrease the risk of air pollutant–related health effects (U.S. EPA 2013).

Populations can experience increased risk for air pollutant–related health effects at a given concentration as a result of multiple avenues, specifically *a*) intrinsic factors, *b*) extrinsic factors, and/or *c*) increased dose (Samet 2011). Intrinsic factors are often defined as individual characteristics that may increase risk through a biological mechanism (e.g., age, sex, genetics), whereas extrinsic factors represent external, nonbiological factors, such as socioeconomic status (SES) or access to health care. Some portions of the population may be at increased risk of an air pollutant–related health effect due to increased internal dose at a given exposure concentration. In addition, populations may be at increased risk of an air pollutant–induced health effect due to differential exposure as a result of, for example, being subjected to higher concentrations of an air pollutant through occupations requiring outdoor work, residential locations near areas of higher concentration, or the lack of household air conditioning units to reduce indoor O_3_ concentrations (Samet 2011). Factors that modify the risk of air pollutant–related health effects may be multifaceted, resulting in a population being at increased or decreased risk because of more than one of these components.

Identifying populations at increased or decreased risk of an air pollutant–related health effect requires defining the attributes of a population that could render them at increased or decreased risk. Previous reviews, such as Sacks et al. (2011), have introduced the idea of using an all-encompassing term, such as “susceptible,” to shift the emphasis away from classifying factors that modify risk into groups such as “susceptible” or “vulnerable” because those terms have been used inconsistently across the literature. This approach, therefore, allows for the focus of any evaluation to be on the fundamental question “What populations are at greatest risk and what evidence forms the basis of this conclusion?” instead of on the categorization of factors. Over time, it has become evident that even the term “susceptible” has underlying connotations and does not accurately capture the entirety of the factors that could modify the risk of an air pollutant–related health effect. As such, we introduce the term “response-modifying factor” (RMF), which we define as any condition or state that alters the exposure or response from an environmental pollutant. RMFs can include intrinsic factors, extrinsic factors, factors that result in differences in dose, and/or factors that result in differential exposure.

The focus on the term “response” characterizes the fact that the studies evaluated to determine the factors that increase or decrease risk are not all epidemiologic studies focusing on a change in the relative risk associated with a health effect; they also include experimental studies that focus on understanding whether biological responses are changing as a result of air pollutant exposures.

Using the approach originally detailed by Sacks et al. (2011) to integrate evidence across the scientific disciplines and determine whether specific populations or life stages are RMFs, in this review we define, evaluate, and characterize the factors that potentially modify the risk of O_3_-related health effects, regardless of whether the increased or decreased risk is due to intrinsic factors, extrinsic factors, increased dose, differential exposure, or a combination of these. To capture the breadth of information on various potential RMFs, we present overviews of each factor instead of detailed reviews. We used a weight-of-evidence approach to draw conclusions with regard to the level of confidence that a specific factor increases or decreases the risk of an O_3_-related health effect. By applying this approach, we were able to identify which factors have the strongest evidence to support their status as RMFs. Our conclusions can be used as the scientific basis for future policy decisions intending to protect those portions of the population at greatest risk, as mandated by the Clean Air Act Amendments of 1990.

## Methods

*Literature search*. To evaluate RMFs that may result in a population or life stage being at increased or decreased risk of an O_3_-related health effect, we focused on the collective evidence evaluated in the most recent scientific review of the O_3_ NAAQS as presented in the 2013 *Integrated Science Assessment* (ISA) *for Ozone and Related Photochemical Oxidants* (U.S. EPA 2013). The O_3_ ISA builds on the conclusions of previous O_3_ assessments (e.g., U.S. EPA 1996, 2006b) and consists of a comprehensive review of papers published in the peer-reviewed literature through July 2012 (with the bulk of the studies published from June 2009 through July 2012) that focused on health effects of short-term (i.e., < 1 month) and long-term (i.e., months to years) O_3_ exposure. These scientific studies were originally identified using an exhaustive literature search strategy in PubMed (http://www.ncbi.nlm.nih.gov/pubmed/) and Web of Science (http://thomsonreuters.com/thomson-reuters-web-of-science/) using the terms “ozone,” “O_3_,” “smog,” and “photochemical oxidant(s)” (retrieving approximately 22,000 references). The broad pollutant-based search was supplemented by various targeted search strategies to identify studies that examined specific health end points. These more targeted searches were determined based on prior knowledge of the key health end points related to O_3_ exposure (i.e., respiratory morbidity, cardiovascular morbidity, mortality, reproductive and developmental effects, and cancer) as well as emerging effects in air pollution literature. The literature search strategy is described in detail in Supplemental Material, “Literature search strategy.”

*Overall study selection and evaluation of study quality*. Once the entire body of scientific literature that examined the effect of O_3_ exposure on various health effects was identified, the U.S. EPA followed a detailed study selection process to identify those references most relevant (i.e., policy relevant) to the O_3_ NAAQS review and evaluate their overall quality (see Supplemental Material, “Study selection and evaluation of individual study quality”). Policy-relevant and informative studies include those that provide a basis for or describe the relationship between O_3_ exposure and effects, including studies that offered innovative methods or design and studies that reduced uncertainty on critical issues. Emphasis was placed on studies that examined effects associated with O_3_ concentrations relevant to current population exposures, and particularly those pertaining to O_3_ concentrations currently found in ambient air. However, studies with higher concentrations were included if they contained unique data, such as a previously unreported biological effect or mode of action or if they examined multiple O_3_ concentrations to elucidate exposure–response relationships. After selecting studies for inclusion, the individual study quality was evaluated by considering the design, methods, and documentation of each study, but not whether the results were positive, negative, or null. This systematic approach to evaluating the literature has been used during the reviews of ISAs for all of the criteria pollutants, including O_3_, which undergo extensive review by an independent panel of subject matter experts, the Clean Air Scientific Advisory Committee.

*Selection of RMF studies*. Of the large body of policy-relevant references (approximately 2,200 studies) that examined the relationship between O_3_ exposure and health effects and were included in the 2013 O_3_ ISA, for this overview, we focused on a subset of studies that contained information on whether specific factors modified the O_3_-associated health response. Details on this approach have been reported previously by Sacks et al. (2011). Briefly, the focus was placed on studies that conducted stratified analyses (e.g., males vs. females) because these studies allowed for a comparison between populations within the same study design. We also evaluated experimental studies (toxicologic and controlled human exposure) to inform coherence with the health effects observed in epidemiologic studies as well as to provide an understanding of biological plausibility. Finally, we included those studies that examined RMFs that may result in differential air pollutant exposures and subsequently a greater risk of O_3_-related health effects in a specific subset of the population, such as studies of outdoor workers.

*Evaluation and characterization of scientific evidence*. For each RMF, the scientific evidence from each study evaluated was integrated across the scientific disciplines (i.e., epidemiologic, controlled human exposure, toxicologic, and exposure sciences studies). It is through this integration that we applied the aspects described by Sir Austin Bradford Hill (Hill 1965)—which included consistency within a discipline, coherence across disciplines, and biologically plausibility—to assess whether a specific factor resulted in a population or life stage being at increased or decreased risk (see Supplemental Material, Table S1). When evaluating the collective evidence for a specific RMF, although the interpretation of individual studies is important, we focused on the overall pattern of effects across studies. For epidemiologic studies, effect measure modification was not necessarily deemed to be present if one comparison group had statistically significant findings while the other group did not. The evaluation of each study included in this overview focused on the examination of the magnitude, direction, and precision of the effect. Evidence of effect measure modification was noted when two comparison groups had different point estimates, regardless of whether the point estimates were statistically significantly different, as well as the degree of confidence interval overlap.

The weight-of-evidence approach we used to assess whether specific factors modify the air pollutant (i.e., O_3_)–health effect association is based on the causal framework developed by the U.S. EPA to evaluate the causal nature of air pollution–related health or welfare effects and used in the ISAs (see Supplemental Material, Table S2) (U.S. EPA 2013). Using this causal framework as a basis, we applied a weight-of-evidence approach, which was also used in the 2013 O_3_ ISA (U.S. EPA 2013), to determine the level of confidence that a specific factor affects the risk of an air pollutant–related health effect. The weight of evidence and considerations underlying each level of classification are presented in Table 1.

**Table 1:** Classification of evidence for potential RMFs.

Level of evidence	Criteria	Potential RMFs
Adequate evidence	There is substantial, consistent evidence within a discipline to conclude that a factor results in a population or life stage being at increased or decreased risk of air pollutant–related health effect(s) relative to some reference population or life stage. Where applicable, this includes coherence across disciplines. Evidence includes multiple high-quality studies.	Genetic factors, asthma, children, older adults, diet, outdoor workers
Suggestive evidence	The collective evidence suggests that a factor results in a population or life stage being at increased or decreased risk of an air pollutant–related health effect relative to some reference population or life stage, but the evidence is limited due to some inconsistency within a discipline or, where applicable, a lack of coherence across disciplines.	Sex, SES, obesity
Inadequate evidence	The collective evidence is inadequate to determine whether a factor results in a population or life stage being at increased or decreased risk of an air pollutant–related health effect relative to some reference population or life stage. The available studies are of insufficient quantity, quality, consistency, and/or statistical power to permit a conclusion to be drawn.	Influenza/infection, chronic obstructive pulmonary disease, cardio­vascular disease, diabetes, hyperthyroidism, race/ethnicity, smoking, air conditioning use
Evidence of no effect	There is substantial, consistent evidence within a discipline to conclude that a factor does not result in a population or life stage being at increased or decreased risk of air pollutant–related health effect(s) relative to some reference population or life stage. Where applicable, this includes coherence across disciplines. Evidence includes multiple high-quality studies.	None identified

In this overview we focus on those RMFs with sufficient evidence (i.e., adequate and suggestive) to draw a conclusion using the weight-of-evidence approach discussed above. We begin this overview by evaluating an RMF that has a strong biological component (genetic factors) and go through a range of potential RMFs, ending with one exclusively related to exposure (i.e., working outdoors). Details of each study are provided in Tables 2 and 3, and in Supplemental Material, Tables S3–S5. An evaluation of all of the RMFs resulted in some factors being deemed to have inadequate evidence but none of the factors had evidence of no effect. These factors are not discussed in this overview, but they are listed in Table 1.

**Table 2:** Summary of results from epidemiologic and controlled human exposure studies of modification by genetic variants: O_3_-related health effects.

Gene variant	Comparison group	Health outcome/population	O_3_ exposure/effect modification of association for the gene variant	Reference
*GSTM1*-null	*GSTM1*-positive	Lung function among healthy adults with intermittent moderate exercise	0.4 ppm, 2 hr; at 24-hr post­exposure, both groups had decreased FEV_1_ and FVC, with no reported difference between the groups.	Alexis et al. 2009
		Inflammatory changes among healthy adults with intermittent moderate exercise	0.4 ppm, 2 hr; at 24-hr post­exposure, *GSTM1*-null had increases in PMN number, oxidative burst, phagocytic function, expression of CD14 on airway PMNs, expression of HLA-DR on airway dendritic cells, expression of HLA-DR on macrophages, and IL-1β and IL-8 compared with *GSTM1*-positive.	
*GSTM1*-null	*GSTM1*-positive	Lung function among healthy adults with intermittent moderate exercise	0.06 ppm, 6.6 hr; both groups had decreased FEV_1_ and FVC, with no reported difference between the groups; no difference was observed for symptom scores between the two groups.	Kim et al. 2011
		Inflammatory responses among healthy adults with intermittent moderate exercise	0.06 ppm, 6.6 hr; both groups had increased percentage of PMNs.	
*GSTM1*-null	*GSTM1*-positive	Respiratory symptoms among children with asthma	8-hr maximum air concentration, 69 ± 31 ppb (mean ± SD); both groups had decreased FEV_1_ and FVC, with no reported difference between the groups, and no difference was observed for symptom scores between the two groups.	Romieu et al. 2006
*GSTM1*-null	*GSTM1*-positive	Lung function among children with asthma	1-hr maximum air concentration, 102 ± 47 ppb (mean ± SD); *GSTM1*-null had decreased FEF_25–75_ compared with *GSTM1*-positive in the placebo group, but no difference was observed between genotypes for the group supplemented with antioxidants.	Romieu et al. 2004b
*GSTP1* Ile/Val or Val/Val	*GSTP1* Ile/Ile	Lung function among adults	2-day air concentration, 24.4 ± 11 ppb (mean ± SD); FEV_1_ was decreased for *GSTP1* Ile/Val or Val/Val compared with Ile/Ile; FVC was possibly decreased as well, but confidence interval overlap was present.	Alexeeff et al. 2008
*GSTP1* Val/Val	*GSTP1* Ile/Ile or Ile/Val	Respiratory symptoms among children with asthma	8-hr maximum air concentration, 69 ± 31 ppb (mean ± SD); *GSTP1* Val/Val had increased difficulty breathing, increased bronchodilator use, and increased cough compared with *GSTP1* Ile/Ile or Ile/Val.	Romieu et al. 2006
*GSTP1* Ile/Ile or Ile/Val	*GSTP1* Val/Val	Lung function among children with asthma	8-hr maximum air concentration, 69 ± 31 ppb (mean ± SD); decreased FEV_1_ and FVC possibly decreased for *GSTP1* Ile/Ile or Ile/Val compared with Val/Val, but the null was included in all estimates.	Romieu et al. 2006
*HMOX1* S/L or L/L	*HMOX1* S/S	Lung function among adults	2-day air concentration, 24.4 ± 11 ppb (mean ± SD); FEV_1_ and FVC were possibly decreased for *HMOX1* S/L or L/L compared with S/S, but confidence interval overlap was present.	Alexeeff et al. 2008
*NQO1*-wildtype and *GSTM1*-null	Other combinations^*a*^	Lung function among healthy adults with exercise	Median 2-hr air concentration, 78 ppb; FEV_1_ was decreased for *NQO1*-wildtype and *GSTM1*-null compared with other combinations; similar FVC changes were observed in both groups.	Bergamaschi et al. 2001
*NQO1*-wildtype and *GSTM1*-null	Other combinations^*a*^	Lung function among mild-to-moderate asthmatics with moderate exercise	0.3 ppm O_3_, 2 hr; no difference was observed in FEV_1_ between the groups.	Vagaggini et al. 2010
		Inflammatory responses among mild-to-moderate asthmatics with moderate exercise	0.3 ppm, 2 hr; quantitative results were not provided, but authors stated that “no difference in the inflammatory response for ozone exposure was found” between the two groups; the researchers counted inflammatory cells including macrophages, lymphocytes, neutrophils, eosinophils, and IL-8.	
Abbreviations: FEF_25–75_, forced expiratory flow 25–75%; FEV_1_, forced expiratory volume in 1 sec; FVC, forced vital capacity; L, long repeats (*n* ≥ 25); PMN, polymorphonuclear neutrophil; S, short repeats (*n* < 25). ^***a***^For example, *NQO1*-defective and *GSTM1*-positive/null.				

**Table 3:** Summary of results from animal toxicology studies of modification by genetic variants: O_3_-related health effects.

Gene variant	Reference	O_3_ exposure	Health outcome
*Cd44*	Garantziotis et al. 2009	2.0 ppm, 3 hr	Decreased AHR
*Csb*	Kooter et al. 2008	1.0 ppm, 3 hr	Decreased BALF TNF-α; no genotype effect on neutrophilia or epithelial damage
*Cxcr2*	Johnston et al. 2005a	1.0 ppm, 3 hr	Decreased neutrophilia, epithelial injury, and AHR; no genotype effect on hyperpermeability
*Hsp70*	Bauer et al. 2011	0.3 ppm, 48 hr	Decreased hyperpermeability and inflammation
*Iai*	Garantziotis et al. 2009	2.0 ppm, 3 hr	Decreased AHR
*Il6*	Johnston et al. 2005b	0.3 ppm, 3 or 72 hr	Decreased soluble TNFR1
*Il6*	Johnston et al. 2005b	0.3 ppm, 72 hr	Decreased hyperpermeability, BALF neutrophils, and soluble TNFR1 and TNFR2; no genotype effect on AHR
*Il6*	Johnston et al. 2005b	2.0 ppm, 3 hr	Reduced BALF neutrophils and soluble TNFR2 and MIP-2
*Il10*	Backus et al. 2010	0.3 ppm, 24, 48, and 72 hr	Increased inflammation; no genotype effect on hyperpermeability
*Il13*	Williams et al. 2008	3.0 ppm, 3 hr	Decreased neutrophilia, BALF cells, and AHR
*Jnk*	Cho et al. 2007	0.3 ppm, 48 hr	Decreased hyperpermeability, neutrophilia, and epithelial damage
*Marco*	Dahl et al. 2007	0.3 ppm, 48 hr	Increased inflammation and hyperpermeability
*Mmp7*	Yoon et al. 2007	0.3 ppm, 48 hr	No genotype effect on hyperpermeability or inflammation
*Mmp9*	Yoon et al. 2007	0.3 ppm, 48 hr	Increased hyperpermeability, neutrophilia, and lung damage
*Myd88*	Williams et al. 2007	3.0 ppm, 3 hr	Decreased inflammation, hyperpermeability, and AHR
*Nfkb1*	Cho et al. 2007	0.3 ppm, 48 hr	Decreased hyperpermeability, neutrophilia, and epithelial damage
*Nos2*	Kleeberger et al. 2001	0.3 ppm, 72 hr	Decreased hyperpermeability; no genotype effect on neutrophilia or epithelial damage
*Nos2*	Fakhrzadeh et al. 2002	0.8 ppm, 3 hr	Decreased hyperpermeability and BALF cells; decreased peroxynitrite, COX1, and COX2 production and increased superoxide anion and PGE_2_ production in alveolar macrophages
*Nos2*	Kenyon et al. 2002	1.0 ppm, 8 hr/night, 3 nights	Increased hyperpermeability and inflammation
*Nqo1*	Voynow et al. 2009	1.0 ppm, 3 hr	Decreased inflammation and AHR
*Tlr2*	Williams et al. 2007	3.0 ppm, 3 hr	Decreased inflammation and AHR; no genotype effect on hyperpermeability
*Tlr4*	Kleeberger et al. 2000	0.3 ppm, 72 hr	Decreased hyperpermeability
*Tlr4*	Hollingsworth et al. 2004	0.3 ppm, 72 hr	No effect on AHR, hyperpermeability, or neutrophilia
*Tlr4*	Hollingsworth et al. 2004	2.0 ppm, 3 hr	Decreased AHR; no genotype effect on hyperpermeability or neutrophilia
*Tlr4*	Williams et al. 2007	3.0 ppm, 3 hr	Decreased AHR and neutrophilia; no genotype effect on hyperpermeability
*Tnfr1/Tnfr2*	Cho et al. 2001, 2007	0.3 ppm, 48 hr	Decreased inflammation and epithelial damage; no genotype effect on hyperpermeability
*Tnfr1/Tnfr2*	Cho et al. 2001, 2007	2.0 ppm, 3 hr	Decreased AHR; no genotype effect on neutrophilia, hyperpermeability, or epithelial damage
Abbreviations: BALF, bronchoalveolar lavage fluid; COX, cytochrome c oxidase subunit; MIP-2, macrophage inflammatory protein-2; Mmp, matrix metallopeptidase; PGE_2_, prostaglandin E receptor 2; Tlr, toll-like receptor; Tnfr, tumor necrosis factor receptor. This table includes animal toxicology studies in which responses were assessed after gene deletion, with the exception of Kleeberger et al. (2000), who compared *C3H/HeJ* (*Tlr4* mutant) to *C3H/HeOuJ* (*Tlr4* normal) mice.			

## Results and Discussion

*Genetic factors*. Specific genetic factors may affect the risk of health effects related to short- and long-term O_3_ exposures, specifically polymorphisms in already identified candidate genes or in genes whose protein products are thought to be involved in the biological mechanism underlying the health effect of an air pollutant (Sacks et al. 2011). Previous reviews reported glutathione *S*-transferase mu 1 (*GSTM1*) and tumor necrosis factor-α (*TNF*) to have a “potential role … in the innate susceptibility to O_3_” (U.S. EPA 2006b). Table 2 provides a summary of recent effect measure modification findings for genetic variants examined in epidemiologic and controlled human exposure studies of respiratory effects. Because of small sample sizes, many controlled human exposure studies are limited in their ability to test genes with low-frequency minor alleles. Among children with asthma, studies of children with the genetic variant of *GSTM1*-null compared with *GSTM1*-positive have reported increased respiratory symptoms and decreased lung function (Romieu et al. 2004b, 2006). Among healthy adults, studies have reported no effect of *GSTM1* variants on lung function and inconsistent results for inflammatory changes (Alexis et al. 2009; Kim et al. 2011). Studies of glutathione *S*-transferase pi 1 (*GSTP1*) have also reported a decrease in lung function and an increase in respiratory symptoms (Alexeeff et al. 2008; Romieu et al. 2004b, 2006). In controlled human exposure studies of NAD(P)H dehydrogenase, quinone 1 (*NQO1*), lung function among healthy adults was decreased for those with *NQO1*-wildtype and *GSTM1*-null gene variants (Bergamaschi et al. 2001). No difference was observed among study participants with asthma (Vagaggini et al. 2010). A study of heme oxygenase (decycling) 1 (*HMOX1*) reported a potential decrease in lung function among adults (Alexeeff et al. 2008).

Toxicologic studies have reported differences in effects after O_3_ exposure among different inbred strains of mice, which indicates that genetic background contributes to differential risk (Chuang et al. 2009; Hamade and Tankersley 2009; Hamade et al. 2008; Tankersley et al. 2010). Inbred strains have been used in genetic linkage and genome-wide association studies to identify candidate genes that lead to increased risk (Cho and Kleeberger 2007), and additional studies have been conducted to validate these candidate genes and other related genes, primarily using mice with targeted gene deletions. Table 3 summarizes recent toxicologic studies that examined the role of gene variants in the modification of the biological response to O_3_ exposure. Overall, these studies show that genes related to innate immune signaling—in particular TNF receptors 1/2 and toll-like receptors 2/4—may modulate risk related to O_3_ exposure, as well as associated genes including *Nfkb1* (nuclear factor of kappa light polypeptide gene enhancer in B cells 1), *Jnk1* (mitogen-activated protein kinase 8; *Mapk8*), *Cd44* (Cd44 antigen), *Myd88* (myeloid differentiation primary response gene 88), *Iai* (inter-α-trypsin inhibitor), *Hsp70* (heat shock protein 70), *Mmp9* (matrix metallopeptidase 9), and *Nos2* (nitric oxide synthase 2, inducible) (Bauer et al. 2011; Cho et al. 2001, 2007; Fakhrzadeh et al. 2002; Garantziotis et al. 2009; Hollingsworth et al. 2004; Kenyon et al. 2002; Kleeberger et al. 2000, 2001; Williams et al. 2007; Yoon et al. 2007). There is also toxicologic evidence indicating that genes involved in pro- and anti-inflammatory signaling and oxidative stress modulate the O_3_ response, including interleukins *Il10*, *Il13*, and *Il6*, and *Cxcr2* [chemokine (C-X-C motif) receptor 2], *Marco* (macrophage receptor with collagenous structure), *Csb* (excision repair cross-complementation group 6), and *Nqo1* (Backus et al. 2010; Dahl et al. 2007; Johnston et al. 2005a, 2005b; Kooter et al. 2008; Voynow et al. 2009; Williams et al. 2008). Taken together, this evidence suggests the complexity of the biological mechanisms underlying airway inflammation and airway hyperresponsiveness (AHR) as well as genetic susceptibility, as previously described by Bauer and Kleeberger (2010).

Collectively, controlled human exposure and epidemiologic studies have reported evidence of O_3_-related increases in respiratory symptoms or decreases in lung function with gene variants, including *GSTM1*, *GSTP1*, *HMOX1,* and *NQO1*. Toxicologic studies of *Nqo1*-deficient mice reported that the mice were resistant to O_3_-induced AHR and inflammation, providing biological plausibility for the results of studies in humans. In addition, studies of rodents have identified a number of other genes that may affect O_3_-related health outcomes, including genes related to innate immune signaling, inflammation, and oxidative stress, which have not been investigated in human studies. Overall, there is “adequate evidence” to indicate that certain genetic variants increased the risk of O_3_-related health effects.

*Life stage*. The 2010 U.S. Census reported that 27.0% of the U.S. population was < 20 years of age, with 13.1% under the age of 10 (Howden and Meyer 2011). In addition, the number of older Americans (i.e., ≥ 65 years of age) is projected to increase from 12.4% to 19.7% of the U.S. population between 2000 and 2030 (U.S. Census Bureau 2010). Therefore, these life stages represent a large population that may potentially be at increased risk of O_3_-related health effects.

Both children and older adults are often considered to be intrinsically at increased risk of O_3_-related health effects because of biological differences compared with the adult population. In children, the respiratory system continues to grow until 18–20 years of age (U.S. EPA 2006b). Young children also have greater lung regional extraction of O_3_, which is thought to be due to smaller nasal and pulmonary region surface areas compared with the total airway surface area in adults (Sarangapani et al. 2003). Children have greater O_3_ tissue doses in the lower airways due to higher ventilation rates per lung volume and a greater oral breathing contribution than adults (Becquemin et al. 1999; Bennett et al. 2008; James et al. 1997). In addition, children often have higher exposure to O_3_ than adults because children tend to spend more time outdoors (Klepeis et al. 1996; U.S. EPA 2011a, 2013). Similar to children, older adults spend slightly more time outdoors than adults 18–64 years of age. However, older adults have somewhat lower ventilation rates than adults 31–60 years of age. The gradual decline in physiologic processes that occur with aging may lead to an increased risk of O_3_-related health effects in older adults (U.S. EPA 2006a).

Controlled human exposure studies have reported that children and adolescents appear, on average, to have nearly equivalent spirometric responses to O_3_ exposure, but they have greater responses than middle-aged and older adults (U.S. EPA 1996). Symptomatic responses (e.g., cough, shortness of breath, pain on deep inspiration) to O_3_ exposure, however, increase with age until early adulthood, and then gradually decrease with increasing age (McDonnell et al. 1999; U.S. EPA 1996). As a result, decreased symptomatic responses may put children and older adults at increased risk because they may withstand continued O_3_ exposure and thus not avoid exposure. In addition, compared with younger age groups, older adults have a higher prevalence of preexisting diseases, with the exception of asthma, and this may also lead to an increased risk of O_3_-related health effects.

Epidemiologic studies have reported greater relative risks for O_3_-related respiratory hospital admissions (HAs) and emergency department (ED) visits among children compared with adults (studies varied with adults defined as all ages > 15 or 18 years or 15–65 years of age) (Halonen et al. 2009; Middleton et al. 2008; Silverman and Ito 2010). However, some studies have reported positive associations among both children and adults, with no evidence of effect measure modification by age (Ko et al. 2007; Mar and Koenig 2009; Paulu and Smith 2008).

The majority of multicity studies that presented age-stratified results—conducted in the United States (Medina-Ramón and Schwartz 2008; Zanobetti and Schwartz 2008), Chile (Cakmak et al. 2007, 2011), and Italy (Stafoggia et al. 2010)—as well as in single-city studies (e.g., Kan et al. 2008) found a trend of increased risk estimates for mortality due to short-term O_3_ exposure in older adults (≥ 65 years of age) compared with younger age groups. Exceptions include the Air Pollution and Health: a European and North American Approach (APHENA) (Katsouyanni et al. 2009), which found increased percent change in mortality risk in the population ≥ 75 years of age in only one study location (i.e., Canada), and a study conducted in Finland (Halonen et al. 2009), which found no evidence of an increased relative risk in the population ≥ 65 years of age compared with the population < 65 years of age. Some of the studies that reported evidence of an increased relative risk in older adults have shown inconsistent results when focusing on individuals who were > 85 years of age, with the relative risk being higher in some cases (Stafoggia et al. 2010) and lower in others (Cakmak et al. 2007). A limited number of epidemiologic studies have examined potential differences in the relative risk by age in studies of respiratory-related HAs and ED visits (Arbex et al. 2009; Halonen et al. 2009) and studies of cardiovascular-related HAs (Buadong et al. 2009; Halonen et al. 2009) and have reported generally inconsistent results. For the studies of cardiovascular-related HAs, results within the general population have been inconsistent and often null; therefore, it is plausible that no association would be observed regardless of age (U.S. EPA 2013).

Toxicologic studies have provided coherence for the potential increased relative risk of O_3_-related health effects by age as demonstrated in epidemiologic studies. Early-life O_3_ exposures of multiple species of laboratory animals, including infant monkeys and rodents, resulted in changes in conducting airways (e.g., Auten et al. 2009; Carey et al. 2007; Fanucchi et al. 2006; Harkema et al. 1987; López et al. 2008; Plopper et al. 2007). In addition, evidence indicates differences in inflammatory responses between neonatal and adult mice (Bils 1970; Vancza et al. 2009). Toxicologic studies have also shown that oxidative damage and stress may be higher after O_3_ exposure in young compared with adult rodents (Fortino et al. 2007; Servais et al. 2005). In addition, a series of studies reported an association between O_3_ exposure and bradycardia that was present among young but not older mice (Hamade et al. 2010; Hamade and Tankersley 2009; Tankersley et al. 2010). Physiologic changes specific to older adults that have been observed in toxicologic studies include changes in heart structure (i.e., ventricular posterior wall thickness at end systole) (Tankersley et al. 2010), wound closure (Lim et al. 2006), and neurodegenerative diseases (as measured by higher lipid peroxidation in the hippocampus) (Rivas-Arancibia et al. 2000).

Generally, epidemiologic studies reported larger associations for respiratory HAs and ED visits for children than adults. However, the interpretation of these studies is limited by the lack of consistency in comparison age groups and in the outcomes examined. For older adults, epidemiologic studies are primarily limited to those examining short-term O_3_ exposure and mortality, but they provide evidence of consistent positive associations in older adults when compared with younger age groups. These results are supported by toxicologic studies that reported effects in younger (i.e., morphologic changes to lung structure) and older animals (i.e., physiologic changes). Also, children and older adults may experience increased exposure due to differences in time spent outdoors, lung regional extraction of O_3_ (children), and ventilation rates as well as a reduction in physiologic response to O_3_ exposures with increasing age. Overall, there is “adequate evidence” indicating that certain life stages (children and older adults) are at increased risk for O_3_-related health effects.

*Sex*. Epidemiologic studies that examined potential differences by sex in associations between O_3_ exposure and respiratory HAs have not consistently found larger relative risk estimates in one group compared with another (Cakmak et al. 2006b; Middleton et al. 2008). For example, there is evidence for higher relative risk estimates in females compared with males in studies of chronic obstructive pulmonary disease HAs and ED visits (Arbex et al. 2009; Wong et al. 2009). However, in studies of asthma ED visits, differences between males and females were observed by age: Larger relative risk estimates were reported for males 2–14 years and females 15–34 years of age, with no evidence of any sex differences in those 35–64 years of age (Paulu and Smith 2008). These results are consistent with Thaller et al. (2008), who found evidence of decreased lung function in females compared with males 16–27 years of age. In addition, Lin et al. (2005) found no evidence for differences in males and females when examining respiratory infection–related HAs in individuals < 15 years of age.

A number of epidemiologic studies that examined cardiovascular-related HAs and ED visits reported no effect modification by sex, with some studies reporting null associations for both males and females (Henrotin et al. 2007; Middleton et al. 2008; Villeneuve et al. 2006; Wong et al. 2009) and one study reporting positive associations for both sexes (Cakmak et al. 2006a). However, the lack of evidence for effect measure modification by sex may be indicative of the lack of association with cardiovascular morbidity, not the lack of an effect by sex (U.S. EPA 2013).

A few epidemiologic studies have examined the association between short-term O_3_ exposure and mortality stratified by sex and, in contrast with studies of other end points, the evidence was more consistent in reporting elevated relative risks among females. These studies, conducted in the United States (Medina-Ramón and Schwartz 2008), Italy (Stafoggia et al. 2010), and Asia (Kan et al. 2008), reported larger effect estimates in females than in males, with some evidence of the relative risk of mortality among females being larger, specifically among those ≥ 60 years of age (Medina-Ramón and Schwartz 2008). However, another study did not find any difference in the relative risk of O_3_-related mortality among men and women (Cakmak et al. 2011).

Experimental studies have described biologically plausible mechanisms that may explain differential risk in O_3_-related health effects between males and females; however, some uncertainty remains. Several controlled human exposure studies have suggested that physiologic differences between the sexes may predispose females to greater effects from O_3_. Specifically, in females, lower plasma and nasal lavage fluid levels of uric acid, the initial defense mechanism of O_3_ neutralization, may result in females being at increased risk of O_3_-related health effects (Housley et al. 1996). Consequently, reduced absorption of O_3_ in the upper airways of females may promote its deeper penetration. In a toxicologic study, Vancza et al. (2009) found small differences in effects by sex in adult mice with respect to pulmonary inflammation and injury after O_3_ exposure, with adult female mice generally more at risk. However, these differences were strain dependent, with some mouse strains exhibiting greater risk in males. The most obvious sex difference in that study was in lactating females, which incurred the greatest lung injury or inflammation among several of the mouse strains. However, not all studies have found differences in the physiologic response to O_3_ exposure. In a controlled human exposure study, Hazucha et al. (2003) reported that forced expiratory volume in 1 sec (FEV_1_) responses in young, healthy females appeared comparable to the response of young males. When evaluating the potential for sex differences in O_3_ absorption in humans, Bush et al. (1996) reported that the absorption distribution of O_3_ was independent of sex when absorption was normalized to anatomic dead space.

Epidemiologic studies of O_3_ exposures and mortality found evidence of elevated relative risks in females, whereas studies of respiratory morbidity found inconsistent results, with some evidence of differences in relative risk by sex depending on age. Although experimental studies provide potential biological plausibility for potential differences by sex, these studies have not consistently demonstrated a clear difference in O_3_-related effects by sex and could potentially be explained by differences in anatomic dead space volume. As a result there is “suggestive evidence” for differences in risk by sex across disciplines.

*Asthma*. In 2008 in the United States, approximately 7.3% of adults and 9.5% of children reported currently having asthma (Bloom et al. 2009; Pleis et al. 2009). As a result, disproportionate effects of O_3_ exposure on the population of individuals with asthma could result in a significant public health impact.

Epidemiologic studies have not consistently demonstrated decreased lung function in asthmatics compared with nonasthmatics in response to short-term O_3_ exposure (Thaller et al. 2008). However, there is some evidence of increased relative risks for wheeze and cough among asthmatics but not nonasthmatics, although this may have been the result of a small nonasthmatic population in this study (Escamilla-Nuñez et al. 2008). Greater short-term, O_3_-associated decreases in lung function have been observed in older individuals with AHR, a sign of asthma, compared with those without AHR (Alexeeff et al. 2007). Further, short-term O_3_ exposure has been reported to be associated with airway inflammation in children regardless of their asthmatic status (Barraza-Villarreal et al. 2008; Berhane et al. 2011). The inconsistency in results across these epidemiologic studies could be due to the studies not accounting for behavioral responses. Recently, Neidell and Kinney (2010) reported that not taking into account individual behavioral adaptations to forecasted air pollution levels (such as avoidance and reduced time outdoors) can underestimate observed associations between short-term O_3_ exposures and respiratory effects.

Similar to the evidence from epidemiologic studies, controlled human exposure studies comparing asthmatics to healthy controls have reported that subjects with asthma appear to be at least as sensitive to the acute effects of O_3_ in terms of FEV_1_ and inflammatory responses as healthy nonasthmatic subjects. According to multiple studies, asthmatics experience greater O_3_-related FEV_1_ decrements than healthy study subjects (Alexis et al. 2000; Horstman et al. 1995; Jorres et al. 1996; Kreit et al. 1989). However, Mudway et al. (2001) reported that individuals with asthma had smaller O_3_-related FEV_1_ decrements than healthy subjects, although the asthmatics in that study tended to be older than the healthy subjects, which could partially explain their smaller response because FEV_1_ responses to O_3_ exposure have been shown to diminish with age. Controlled human exposure studies have also reported subclinical changes in individuals with asthma (compared with similarly exposed healthy individuals) including increased neutrophils in bronchoalveolar lavage fluid, higher levels of cytokines and hyaluronan in lavage fluid or sputum, and greater expression of macrophage cell-surface markers, which provide biological plausibility for the increased O_3_-related health effects observed in asthmatics (Basha et al. 1994; Bosson et al. 2003; Hernandez et al. 2010; Peden et al. 1997; Scannell et al. 1996).

Toxicologic studies are coherent with other studies showing greater O_3_ effects among those with asthma or AHR. Using an asthmatic phenotype modeled by allergic sensitization of the respiratory tract, effects of O_3_ on pulmonary function have been found to be augmented by allergic sensitization in infant rhesus monkeys (Fanucchi et al. 2006; Joad et al. 2006; Schelegle et al. 2003), mice (Funabashi et al. 2004), and rats (Wagner et al. 2007). In addition, in a bleomycin-induced pulmonary fibrosis rat model, exposure to O_3_ increased pulmonary inflammation and fibrosis, along with the frequency of bronchopneumonia (Oyarzún et al. 2005). Thus, short-term O_3_ exposure may also enhance damage in a previously injured lung.

Epidemiologic studies demonstrate that asthmatics are at least as sensitive as healthy individuals to O_3_-related health effects. Controlled human exposure studies have shown increased FEV_1_ decrements and inflammatory responses in asthmatics compared with healthy individuals. Finally, controlled human exposure and toxicologic studies using animal models of asthma provide biological plausibility for the effects observed in some epidemiologic studies. Overall, there is “adequate evidence” that people with asthma are at increased risk of O_3_-related health effects.

*Obesity*. Obesity, defined as a body mass index (BMI) of ≥ 30 kg/m^2^, is an issue of increasing importance in the United States, with self-reported rates of obesity of 26.7% in 2009, up from 19.8% in 2000 (Sherry et al. 2010). Recent studies of air pollution have begun to examine whether obesity is a risk factor for air pollution–related health effects. In an epidemiologic study, Alexeeff et al. (2007) reported decreased lung function with increased short-term O_3_ exposure for both obese and nonobese subjects; however, the magnitude of the reduction in lung function was greater for obese individuals. These authors noted further decrements in lung function for obese individuals who also had AHR.

Controlled human exposure studies have detected differential effects of O_3_ exposure on lung function for individuals with varying BMIs. In a retrospective analysis of data from healthy, nonsmoking, white males 18–35 years of age, McDonnell et al. (2010) observed that increased BMI was associated with enhanced FEV_1_ responses. In a similar analysis, Bennett et al. (2007) observed greater O_3_-related FEV_1_ decrements with increasing BMI in a group of healthy, nonsmoking women (BMI, 15.7–33.4 kg/m^2^) but not among healthy, nonsmoking men (BMI, 19.1– 32.9 kg/m^2^), indicating that sex may also play a role in any O_3_ effects attributed to obesity.

Animal toxicologic studies have also reported enhanced pulmonary inflammation and injury with acute O_3_ exposure in genetic and diet-induced obese mice, providing biological plausibility and coherence with the effects observed in epidemiologic and controlled human exposure studies (Johnston et al. 2008; reviewed by Shore 2007). However, a recent study (Shore et al. 2009) reported that obese mice were resistant to O_3_-related pulmonary injury and inflammation and reduced lung compliance after longer exposures at lower concentrations, regardless of whether obesity was genetically induced or diet induced.

Epidemiologic and controlled human exposure studies have reported evidence for increased O_3_-related respiratory health effects among obese individuals. Toxicologic studies are generally coherent with evidence in epidemiologic and controlled human exposure studies. Some, but not all, studies support the possibility of increased risk of O_3_-related pulmonary effects among obese individuals. Overall, there is “suggestive evidence” that obese individuals are at increased risk of O_3_-related health effects.

*Diet*. Diet, which is strongly correlated with other factors such as obesity and SES, may modify the association between O_3_ exposure and health effects. Ozone mediates some of its toxic effects through oxidative stress (U.S. EPA 2013); therefore, the antioxidant status of an individual is an important factor that may affect the risk of O_3_-related health effects. As a result, a number of studies have examined dietary factors, specifically, supplementation with antioxidant vitamins (e.g., vitamins C and E), to identify whether these factors inhibit O_3_-mediated damage.

In epidemiologic studies, increases in fruit/vegetable intake and Mediterranean dietary patterns, which have been noted for their high content of vitamins C and E and omega-3 fatty acid, have been found to protect against O_3_-related decreases in lung function among children (Romieu et al. 2009). Similarly, the protective effect of dietary supplementation in asthmatic children was demonstrated by an association between short-term O_3_ exposure and nasal airway inflammation among a placebo group but not among a group supplemented with vitamins C and E (Sienra-Monge et al. 2004).

Results from epidemiologic studies are consistent with those observed in controlled human exposure studies that provide evidence of protective effects of α-tocopherol (a form of vitamin E) and ascorbate (vitamin C) on spirometric measures of lung function after O_3_ exposure, but not on the intensity of subjective symptoms and inflammatory response including cell recruitment and activation and release of mediators (Samet et al. 2001; Trenga et al. 2001). Dietary antioxidants have also afforded protection to asthmatics by attenuating postexposure bronchial hyperresponsiveness (Trenga et al. 2001).

Toxicologic studies also provide evidence of protective effects from vitamin supplementation, which is consistent with evidence from epidemiologic and controlled human exposure studies. In rats, γ-tocopherol treatment has been reported to inhibit O_3_-related inflammation and mucus production and to reduce O_3_-exacerbated nasal allergy responses (Wagner et al. 2007, 2009). Similarly, supplementation with vitamins C and E has resulted in attenuation of inflammation, oxidative stress, and AHR in guinea pigs exposed subchronically to O_3_ (Chhabra et al. 2010). However, in another study, guinea pigs deficient in vitamin C displayed only minimal differences in injury and inflammation after exposure to O_3_ compared with vitamin C–sufficient animals (Kodavanti et al. 1995). Additional studies have reported that β-carotene and vitamin A supplementation was protective against the effects of O_3_ exposure (Paquette et al. 1996; Valacchi et al. 2009).

Consistent evidence across disciplines indicates that individuals with reduced intake of vitamins C and E are at increased risk for O_3_-related health effects. The evidence from epidemiologic studies is supported by controlled human exposure and toxicologic studies, and collectively provides “adequate evidence” that individuals with an insufficient diet are at increased risk of O_3_-related health effects.

*SES*. SES is often represented by personal- or neighborhood-level SES, which comprises a variety of components such as educational attainment, household income, and health insurance status. SES is typically indicative of such things as access to health care, quality of housing, and the pollution gradient to which people are exposed.

Multiple epidemiologic studies have reported that individuals of low SES have an increased relative risk of respiratory effects (e.g., HAs and ED visits) due to O_3_ exposures. This includes studies that examined SES using neighborhood-level educational attainment in Canada (Cakmak et al. 2006b) and regional insurance rates in Korea (Lee et al. 2006). However, some studies, specifically in Canada, have found no evidence of modification of the relative risk using measures of neighborhood-level income (Burra et al. 2009; Cakmak et al. 2006b). SES was also examined in a study of short-term O_3_ exposures and cardiac disease ED visits in Canada where neighborhood-level education or income was divided into quartiles (Cakmak et al. 2006a). Cakmak et al. (2006a) did not observe effect measure modification of cardiac disease ED visits by any level of neighborhood education or income, which may not necessarily inform SES differences overall due to the limited evidence for O_3_-induced cardiovascular-related HAs and ED visits in the general population, as mentioned above.

Several large-scale epidemiologic studies [i.e., NMMAPS (the National Morbidity, Mortality, and Air Pollution Study) and APHENA] reported increased relative risk of O_3_-related mortality among groups with lower SES based on neighborhood-level unemployment in the United States (Bell and Dominici 2008; Katsouyanni et al. 2009). Increases in O_3_-related mortality have also been observed in studies using individual-level education, individual-level occupation, and neighborhood-level income as measures of SES (Cakmak et al. 2011). Other studies conducted in China and Italy reported inconsistent or null findings using individual-level educational attainment (Kan et al. 2008), a neighborhood-level deprivation index (Wong et al. 2008), and neighborhood-level income (Stafoggia et al. 2010). The influence of SES on mortality has also been examined in studies of infant mortality in Mexico. These studies found no association between O_3_ concentrations and infant mortality regardless of SES measured using neighborhood-level indicators such as income or availability of public services (Carbajal-Arroyo et al. 2011; Romieu et al. 2004a); however, Carbajal-Arroyo et al. (2011) reported evidence of a positive association for respiratory-related infant mortality in only the low-SES group.

Morello-Frosch et al. (2010) reported greater decreases in birth weight associated with full pregnancy O_3_ concentration for those with higher neighborhood poverty rates. However, a study conducted in Australia using a neighborhood-level SES index composed of multiple factors such as income and unemployment demonstrated no modification of the association between O_3_ exposure during days 31–60 of gestation and abdominal circumference during gestation despite some evidence of an inverse association in the highest SES quartile (Hansen et al. 2008).

A single controlled human exposure study examined O_3_ effects on lung function and potential modification of response among three SES categories (based on father’s educational attainment), although the study was not originally designed to investigate SES (Seal et al. 1996). Individuals in the middle SES category showed a greater concentration-dependent decline in percent predicted FEV_1_ than did the low- and high-SES groups. However, it was unclear why differences were greatest in the middle SES group in that study.

Most studies have reported that individuals with low SES or those living in neighborhoods with low SES have an increased relative risk of O_3_-related respiratory HA and ED visits. Inconsistent results have been observed in the few studies that examined effect measure modification of the O_3_ association with mortality and reproductive outcomes. A controlled human exposure study, although not designed to examine differences by SES, did not support evidence of increased risk of O_3_-related health effects among individuals with lower SES. Overall, there is “suggestive evidence” that individuals of low SES are at increased risk of experiencing O_3_-related health effects.

*Outdoor workers*. Multiple epidemiologic studies have found that individuals who participate in outdoor activities or work outside are a population at increased risk of air pollution–related health effects due to increased exposure, which has been affirmed by studies that have reported consistent associations between O_3_ exposure and respiratory health outcomes in these groups (U.S. EPA 2006b). Outdoor workers are exposed to ambient O_3_ concentrations for a greater period of time than individuals who spend their days indoors. In addition, an increase in dose to the lower airways in this population is expected due to both increases in the amount of air breathed (i.e., minute ventilation) and a shift from nasal to oronasal breathing that traditionally occurs during outdoor exercise (Hu et al. 1994; Nodelman and Ultman 1999). However, the health effects in this population seem to be limited to respiratory-related effects as evidenced by an epidemiologic study exploring effect measure modification of O_3_ exposure by workplace (i.e., indoor/outdoor) on DNA damage, which found inconsistent results (Tovalin et al. 2006).

There is strong evidence demonstrating increased exposure, dose, and ultimately risk of O_3_-related respiratory effects in outdoor workers. Overall, there is “adequate evidence” that outdoor workers are at increased risk of O_3_-related health effects.

*Limitations*. We recognize that, in some cases, it is difficult to clearly determine whether a factor leads to increased or decreased risk of a population experiencing O_3_-related health effects. Not only is this due to inconsistencies within a discipline, the lack of coherence across disciplines, or the lack of biological plausibility but also to intersubject variability and the possible attenuation of O_3_-related effects. Controlled human exposure studies have clearly shown intersubject variability in respiratory-related responses to O_3_ exposure among healthy adults (Holz et al. 2005; McDonnell 1996; Que et al. 2011). These responses tend to be reproducible within a given individual over a period of several months, indicating differences in the intrinsic responsiveness (Hazucha et al. 2003; Holz et al. 1999, 2005; McDonnell et al. 1985). In addition, preexposure to O_3_ has been reported to lead to an attenuation of lung function and symptomatic responses to O_3_ on subsequent days (Foxcroft and Adams 1986).

Inconsistency in the categorization and/or measurement of a factor across studies makes drawing conclusions regarding potential RMFs difficult. For example, numerous metrics are used to characterize SES. In addition, when considering epidemiologic studies conducted in other countries, it should be noted that it is possible those populations may differ in SES or other demographic indicators (e.g., overall health status), thus limiting generalizability to a U.S. population.

Furthermore, there is the possibility of publication bias. Stratified analyses that have interesting effect measure modification results may be presented in publications, whereas studies that find no evidence of effect measure modification may not. It is not possible to measure the influence of publication bias on our overall conclusions; therefore, some of the evidence may be more varied than it appears.

Finally, we recognize that additional studies that could inform the conclusions we reached in our evaluation of the scientific evidence could have been missed in our literature search. We focused on searches using Web of Science and PubMed, and we did not use other databases such as Embase (http://www.elsevier.com/online-tools/embase). In addition, our systematic literature search was limited to recent years, although informative studies included in past assessments were also included. The literature summarized in this overview was drawn from the 2013 O_3_ ISA (U.S. EPA 2013), which was reviewed by scientific experts and had an associated call for papers/public comment. Therefore, we are confident that all relevant papers were captured.

## Conclusions

The integration of evidence across scientific disciplines—which allows for an evaluation of the consistency of effects within and the coherence of effects across disciplines, as well as an evaluation of biological plausibility—provides a scientific basis for drawing conclusions regarding populations that are potentially at increased or decreased risk of an air pollutant–related health effect. Based on our evaluation of the scientific evidence, we concluded that there is “adequate” evidence for increased risk of O_3_-related health effects in population groups with certain genotypes, preexisting asthma, or reduced intake of certain nutrients; individuals at certain life stages (i.e., younger and older ages); and in outdoor workers (Table 1). Other factors (i.e., sex, SES, and obesity) were characterized by “suggestive” evidence for increased risk of O_3_-related health effects.

## Supplemental Material

(2 MB) PDF
