# Supplementary material for: Evaluating Potential Response-Modifying Factors for Associations between Ozone and Health Outcomes: A Weight-of-Evidence Approach
Source: Environ Health Perspect. 2014 Jun 13;122(11):1166–76. doi: 10.1289/ehp.1307541 (PMC4216162; doi:10.1289/ehp.1307541)
Supplement: (2 MB) PDF [file ehp.1307541.s001.508.pdf]

Environmental Health Perspectives doi:10.1289/ehp.1307541

Supplemental Material

Evaluating Potential Response-Modifying Factors for Associations between Ozone and Health Outcomes: A Weight-of-Evidence Approach

Lisa C. Vinikoor-Imler, Elizabeth O. Owens, Jennifer L. Nichols, Mary Ross, James S. Brown, and Jason D. Sack

Table of Contents **Page**

Supplemental Information: Methods **2**
Literature search strategy **2**
Study selection and evaluation of individual study quality **3**
Evaluation of scientific evidence and the causal framework **4**

Table S1. Table from the US EPA's Integrated Science Assessments, "Aspects to aid in judging causality" **6**

Table S2. Weight of evidence for causal determination **7**

Table S3. Results from epidemiologic studies examining response modifying factors for O_3-related health effects **8**

Table S4. Results from controlled human exposure studies examining response modifying factors for O_3-related health effects **21**

Table S5. Results from toxicological studies examining response modifying factors for O_3-related health effects **25**

References **31**

Supplemental Information: Methods

Literature search strategy

The EPA maintains an ongoing literature search process for the identification of relevant scientific studies published since the last review of the National Ambient Air Quality Standard (NAAQS) (U.S. EPA 2011) for each criteria pollutant, including the O_3 Integrated Science Assessment (ISA). For the identification of new studies, search strategies are designed for pollutants and scientific disciplines and iteratively modified to optimize identification of pertinent publications. For this search, the terms “ozone”, “O_3”, “smog”, and “photochemical oxidant(s)” were used in both PubMed and Web of Science. In addition, studies were identified for inclusion in the ISA in several ways: specialized searches on specific topics; independent review of tables of contents for journals in which relevant papers may be published; identification of relevant literature by expert scientists; review of citations in previous assessments and identification by the public and the Clean Air Scientific Advisory Committee (CASAC) (U.S. EPA 2014) during the external review process. Additionally, during the process of developing the O_3 Integrated Science Assessment (ISA), scientific publications are provided to the Agency by the public through a call for information in the Federal Register (U.S. EPA, 2008).

References identified through the multipronged search strategy were screened by title and abstract by scientists at the EPA. Non-English language papers were excluded. Those references that were potentially relevant after reading the title were “considered” for inclusion in the ISA and were added to the Health and Environmental Research Online (HERO) database developed by EPA; which is available to the public.

Only those studies that have undergone scientific peer review and have been published or accepted for publication and published reports that have undergone peer review were considered for inclusion. All relevant epidemiologic, controlled human exposure, toxicological, ecological, and welfare effects studies published since the last O_3 review were considered, including those related to exposure-response relationships, mode(s) of action (MOA), and response modifying factors that may increase or decrease the risk of an O_3-related health effect in specific populations and lifestages. Studies and data analyses on atmospheric chemistry, air quality and emissions, environmental fate and transport, dosimetry, toxicokinetics and exposure were also considered for inclusion. This large global search identified approximately 22,000 studies that examined both health and ecological effects and O_3 exposure.

Study selection and evaluation of individual study quality

After the literature search was conducted the selection of studies considered for inclusion was based on the extent to which the study is informative and policy-relevant. This evaluation was performed by scientists at the EPA for studies of health, ecological, and welfare effects; however for this paper we focus on the identification and evaluation of health effects studies.

In general, in assessing the scientific quality of health effects studies, the following considerations were taken into account.

- Were study design, study groups, methods, data, and results clearly presented to allow for study evaluation?
- Were the study site(s), study populations, subjects, or organism models adequately selected, and are they sufficiently well-defined to allow for meaningful comparisons between study or exposure groups?

- Are the air quality data, exposure, or dose metrics of adequate quality and sufficiently representative of information regarding ambient conditions?
- Are the health effect measurements meaningful, valid and reliable?
- Were likely covariates or modifying factors adequately controlled or taken into account in the study design and statistical analysis?
- Do the analytical methods provide adequate sensitivity and precision to support conclusions?
- Were the statistical analyses appropriate, properly performed, and properly interpreted?

These criteria provide benchmarks for evaluating various studies and for focusing on the policy-relevant studies in assessing the body of health effects evidence. Of most relevance for inclusion are studies that provide useful qualitative or quantitative information on O_3 exposure-effect or exposure-response relationships at doses or concentrations relevant to ambient conditions that can inform decisions on whether to retain or revise the standards. Therefore, concentrations above 2 ppm were excluded from the review.

The results from the large global search were reduced using exclusion criteria (e.g. non-English language and not related to ambient air, such as disinfection byproducts) and targeted searches for key health endpoints to 4,057 references that were considered for inclusion in the O_3 ISA. A total of 2,270 references deemed by EPA scientists to be of high quality, based on the above considerations, was included in the final document.

Evaluation of scientific evidence and the causal framework

To aid judgment in interpreting scientific results, various "aspects" of causality have been discussed by many philosophers and scientists. The "aspects" to judging causality developed by

Sir Austin Bradford Hill (Hill 1965) formed the basis for EPA's causal determination framework, but was modified to encompass a broader array of data (Table S1), i.e., epidemiologic, controlled human exposure, ecological, and animal toxicological studies, as well as in vitro data, and to be more consistent with EPA's Guidelines for Carcinogen Risk Assessment (U.S. EPA 2009; U.S. EPA 2005). Additionally this framework was developed to be specific to examining causality for health and welfare effects for pollutant exposures. Although these aspects provide a framework for assessing the evidence, they do not lend themselves to being considered in terms of simple formulas or fixed rules of the evidence necessary to lead to conclusions about causality (Hill 1965). Rather, these aspects provide a framework for systematic appraisal of the body of evidence, informed by peer and public comment and advice, which includes weighing alternative views on controversial issues. In addition, it is important to note that the aspects presented in Table S1 cannot be used as a strict checklist, but rather to determine the weight of the evidence for inferring causality. In particular, not meeting one or more of the principles does not automatically preclude a determination of causality [see discussion in (CDC 2004)]. Building off these aspects used to judge causality the US EPA developed a causal framework to draw conclusions regarding the causal relationship between relevant pollutant exposures and health or environmental effects as discussed in the O_3 ISA. This weight of evidence approach is detailed in Table S2. It is with these aspects in judging causality in mind that we modified the causality framework detailed in Table S2 to encompass examining response modifying factors, which is used to draw conclusions regarding whether a specific factor increases or decreases the risk of an air pollutant (i.e. O_3)-related health effect.

Table S1. Table from the US EPA's Integrated Science Assessments, "Aspects to aid in judging causality."

Aspect	Description
Consistency of the observed association	An inference of causality is strengthened when a pattern of elevated risks is observed across several independent studies. The reproducibility of findings constitutes one of the strongest arguments for causality. If there are discordant results among investigations, possible reasons such as differences in exposure, confounding factors, and the power of the study are considered.
Coherence	An inference of causality from one line of evidence (e.g., epidemiologic, clinical, or animal studies) may be strengthened by other lines of evidence that support a cause-and-effect interpretation of the association. Evidence on ecological or welfare effects may be drawn from a variety of experimental approaches (e.g., greenhouse, laboratory, and field) and subdisciplines of ecology (e.g., community ecology, biogeochemistry, and paleontological/historical reconstructions). The coherence of evidence from various fields greatly adds to the strength of an inference of causality. In addition, there may be coherence in demonstrating effects across multiple study designs or related health endpoints within one scientific line of evidence.
Biological plausibility.	An inference of causality tends to be strengthened by consistency with data from experimental studies or other sources demonstrating plausible biological mechanisms. A proposed mechanistic linking between an effect and exposure to the agent is an important source of support for causality, especially when data establishing the existence and functioning of those mechanistic links are available.
Biological gradient (exposure-response relationship)	A well-characterized exposure-response relationship (e.g., increasing effects associated with greater exposure) strongly suggests cause and effect, especially when such relationships are also observed for duration of exposure (e.g., increasing effects observed following longer exposure times).
Strength of the observed association	The finding of large, precise risks increases confidence that the association is not likely due to chance, bias, or other factors. However, it is noted that a small magnitude in an effect estimate may represent a substantial effect in a population.
Experimental evidence	Strong evidence for causality can be provided through "natural experiments" when a change in exposure is found to result in a change in occurrence or frequency of health or welfare effects.
Temporal relationship of the observed association	Evidence of a temporal sequence between the introduction of an agent, and appearance of the effect, constitutes another argument in favor of causality.
Specificity of the observed association	Evidence linking a specific outcome to an exposure can provide a strong argument for causation. However, it must be recognized that rarely, if ever, does exposure to a pollutant invariably predict the occurrence of an outcome, and that a given outcome may have multiple causes.
Analogy	Structure activity relationships and information on the agent's structural analogs can provide insight into whether an association is causal. Similarly, information on mode of action for a chemical, as one of many structural analogs, can inform decisions regarding likely causality.

Source: U.S. EPA, 2013.

Table S2. Weight of evidence for causal determination.

	**Health effects**
Causal relationship	Evidence is sufficient to conclude that there is a causal relationship with relevant pollutant exposures (i.e., doses or exposures generally within one to two orders of magnitude of current levels). That is, the pollutant has been shown to result in health effects in studies in which chance, bias, and confounding could be ruled out with reasonable confidence. For example: a) controlled human exposure studies that demonstrate consistent effects; or b) observational studies that cannot be explained by plausible alternatives or are supported by other lines of evidence (e.g., animal studies or mode of action information). Evidence includes multiple high-quality studies.
Likely to be a causal relationship	Evidence is sufficient to conclude that a causal relationship is likely to exist with relevant pollutant exposures, but important uncertainties remain. That is, the pollutant has been shown to result in health effects in studies in which chance and bias can be ruled out with reasonable confidence but potential issues remain. For example: a) observational studies show an association, but copollutant exposures are difficult to address and/or other lines of evidence (controlled human exposure, animal, or mode of action information) are limited or inconsistent; or b) animal toxicological evidence from multiple studies from different laboratories that demonstrate effects, but limited or no human data are available. Evidence generally includes multiple high-quality studies.
Suggestive of a causal relationship	Evidence is suggestive of a causal relationship with relevant pollutant exposures, but is limited. For example, (a) at least one high-quality epidemiologic study shows an association with a given health outcome but the results of other studies are inconsistent; or (b) a well-conducted toxicological study, such as those conducted in the National Toxicology Program (NTP), shows effects in animal species,
Inadequate to infer a causal relationship	Evidence is inadequate to determine that a causal relationship exists with relevant pollutant exposures. The available studies are of insufficient quantity, quality, consistency, or statistical power to permit a conclusion regarding the presence or absence of an effect.
Not likely to be a causal relationship	Evidence is suggestive of no causal relationship with relevant pollutant exposures. Several adequate studies, covering the full range of levels of exposure that human beings are known to encounter and considering at-risk populations, are mutually consistent in not showing an effect at any level of exposure.

Source: U.S. EPA, 2013.

Table S3. Results from epidemiologic studies examining response modifying factors for O_3-related health effects (listed in order of appearance within each section).

Study	Location	Comparison populations	Exposure duration	Health endpoint	Effect estimate	Results
**Lifestage**						
Children						
Halonen et al. 2009	Helsinki, Finland	<15yrs, 15-64 yrs, 65+ yrs	Short-term; warm-season, median 8-h max 35.7 ppb	Asthma ED visits	% change (95% CI) per 12.8 ppb	Asthma hospitalizations: <15 yrs: 12.6 (0.80, 25.8); Asthma-COPD hospitalizations: 15-64 yrs: 3.23 (-6.84, 14.4), 65+ yrs: 9.62 (2.02, 17.8)
Middleton et al. 2008	Nicosia, Cyprus	<15 yrs, >15 yrs	Short-term; mean 8-h max ranged by location and season from 28.7 ppb (12.6 ppb) to 54.9 ppb (8.2 ppb)	All-cause hospitalizations, respiratory hospitalizations	% increase (95% CI) per 10 ppb	All-cause hospitalizations: <15 yrs: 1.58 (0.25, 2.92), >15 yrs: 0.15 (-0.62, 0.92); Respiratory hospitalizations: <15 yrs: 2.27 (-0.95, 5.60), >15 yrs: -1.65 (-4.89, 1.70)
Silverman and Ito 2010	New York City, U.S.	<6 yrs, 6-18 yrs, 19-49 yrs, 50+ yrs	Short-term; median warm season 8-h max 41 ppb	Asthma-related hospitalizations	RR (95% CI) per 22 ppb	ICU hospitalizations: <6 yrs: 0.96 (0.83, 1.11), 6-18 yrs: 1.19 (1.01, 1.40), 19-49 yrs: 1.03 (0.92, 1.16), 50+ yrs: 1.00 (0.90, 1.10); Non-ICU hospitalizations: <6 yrs: 1.09 (1.04, 1.15), 6-18 yrs: 1.20 (1.11, 1.29), 19-49 yrs: 1.08 (1.04, 1.12), 50+ yrs: 1.06 (1.02, 1.10)
Ko et al. 2007	Hong Kong	0-14 yrs, 15-65 yrs, 66+ yrs	Short-term; mean 8-hr avg 21.7 ppb (11.9 ppb)	Asthma-related hospitalizations	RR (95% CI) per 5 ppb	0-14 yrs: 1.039 (1.030, 1.048), 15-65 yrs: 1.041 (1.032, 1.050), 66+ yrs: 1.023 (1.015, 1.030)
Paulu and Smith 2008	Maine, U.S.	2-14 yrs, 15-34 yrs, 35-64 yrs	Short-term; median warm-season 8-hr max ranged by year from 36 ppb to 42 ppb	Asthma-related ED visits	% increase (95% CI) per 10 ppb	2-14 yrs: 11 (1, 23), 15-34 yrs: 16 (8, 24); By Age and Sex: Males: 2-14 yrs: 17 (3, 32), 15-34 yrs: 10, 35-64 yrs: 11; Females: 2-14 yrs: 4, 15-34 yrs: 20 (10, 31), 35-64 yrs: 8
Mar and Koenig 2009	Seattle, Washington, U.S.	0-18 yrs, 18+ yrs	Short-term; mean 1-h max 38.6 ppb	Asthma-related ED visits	RR (95% CI) per 10 ppb	0-18 yrs: 1.07 (0.99, 1.17), 18+ yrs: 1.06 (1.01, 1.12)

Study	Location	Comparison populations	Exposure duration	Health endpoint	Effect estimate	Results
Older adults						
Medina-Ramón and Schwartz 2008	U.S.	<65 yrs, 65+ yrs	Short-term; median warm-season 8-hr avg ranged by city from 16.1 ppb to 58.8 ppb	Mortality	Additional % change in 65+ compared to <65 per 10 ppb	1.10 (95% CI 0.44, 1.77); By Age and Sex (compared to men in the same age groups): Women <60 yrs: -0.09 (-0.76, 0.58), Women 60+ yrs: 0.60 (0.25, 0.96)
Zanobetti and Schwartz 2008	U.S.	0-20 yrs, 21-30 yrs, 31-40 yrs, 41-50 yrs, 51-60 yrs, 61-70 yrs, 71-80 yrs, 81+ yrs	Short-term; mean 8-hr avg ranged by season from 16.5 ppb to 47.8 ppb	Mortality	% increase (95% CI) per 10 ppb	0-20 yrs: 0.08 (-0.42, 0.57), 21-30 yrs: 0.10 (-0.67, 0.87), 31-40 yrs: 0.07 (-0.38, 0.52), 41-50 yrs: 0.08 (-0.27, 0.43), 51-60 yrs: 0.54 (0.19, 0.89), 61-70 yrs: 0.38 (0.16, 0.61), 71-80 yrs: 0.50 (0.32, 0.67), 81+ yrs: 0.29 (0.13, 0.44)
Cakmak et al. 2007	Chile	<64 yrs, 65-74 yrs, 75-84 yrs, 85+ yrs	Short-term; mean 1-h max 100.13 ppb	Mortality	% change (t-ratio) per 100.13 ppb	<64 yrs: 4.96 (1.17), 65-74 yrs: 8.00 (1.77), 75-84 yrs: 9.42 (2.28), 85+ yrs: 8.56 (2.02)
Cakmak et al. 2011	Chile	<64 yrs, 65-74 yrs, 75-84 yrs, 85+ yrs	Short-term; avg 8-h max ozone ranged by city from 59 ppb (SD 56.6 ppb) to 87.6 ppb (86.2 ppb)	Mortality	RR (95% CI) per IQR	<64 yrs: 1.033 (0.993, 1.076), 65-74 yrs: 1.033 (0.982, 1.088), 75-84 yrs: 1.073 (1.014, 1.135), 85+ yrs: 1.063 (1.012, 1.117)
Stafoggia et al. 2010	Italy	35-64 yrs, 65-74 yrs, 75-84 yrs, 85+ yrs	Short-term; mean 8-h moving avg ranged by city from 39.0 ppb (SD 10.3 ppb) to 57.7 ppb (SD 19.3 ppb)	Mortality	% increase (95% CI) per 5 ppb	35-64 yrs: 0.8 (-0.8, 2.5), 65-74 yrs: 0.9 (-0.5, 2.3), 75-84 yrs: 0.5 (-0.5, 1.5), 85+ yrs: 3.5 (2.4, 4.6)
Kan et al. 2008	Shanghai, China	5-44 yrs, 45-64 yrs, 65+ yrs	Short-term; mean 8-h avg 31.7 ppb (SD 0.5 ppb)	Mortality	% increase (95% CI) per 5 ppb	5-44 yrs: -0.08 (-1.38, 1.25), 45-64 yrs: 0.47 (-0.19, 1.12), 65+ yrs: 0.32 (0.03, 0.61)

Study	Location	Comparison populations	Exposure duration	Health endpoint	Effect estimate	Results
Katsouyanni et al. 2009	U.S., Canada, Europe	<75 yrs, 75+ yrs	Short-term; mean 1-h max ranged from 13.3-38.4 ppb in the US, 6.7-8.4 ppb in Canada, and 18.3-41.9 ppb in Europe	Mortality	% change (95% CI) per 5 ppb	All-cause mortality: United States: <75 yrs: 0.45 (0.15, 0.75), 75+ yrs: 0.32 (-0.04, 0.67); Canada: <75 yrs: 0.65 (-0.043, 1.3), 75+ yrs: 0.83 (0.11, 1.6); Europe: <75 yrs: 0.35 (0.12, 0.57), 75+ yrs: 0.11 (-0.10, 0.31); Cardiovascular mortality: United States: <75 yrs: 0.48 (-0.02, 0.98), 75+ yrs: 0.29 (-0.17, 0.75); Canada: <75 yrs: 0.83 (0.11, 1.6), 75+ yrs: 0.65 (-0.043, 1.3); Europe: <75 yrs: 0.25 (-0.14, 0.64), 75+ yrs: 0.26 (-0.03, 0.54)
Halonen et al. 2009	Helsinki, Finland	15-64 yrs, 65+ yrs	Short-term; warm-season, median 8-h max 35.7 ppb	Cardiovascular mortality, respiratory mortality, hospitalizations related to coronary heart disease, stroke, arrhythmia, pneumonia, asthma-COPD	% change (95% CI) per 12.8 ppb	Cardiovascular mortality: 15-64 yrs: 4.44 (-7.85, 18.4), 65+ yrs: 0.67 (-4.57, 6.20); Respiratory mortality: 15-64 yrs: 3.02 (-10.9, 19.1), 65+ yrs: 2.22 (-8.80, 14.6); Coronary heart disease hospitalizations: 15-64 yrs: 0.21 (-6.53, 7.43), 65+ yrs: -1.06 (-5.18, 3.23); Stroke hospitalizations: 15-64 yrs: 1.96 (-5.85, 10.4), 65+ yrs: 2.24 (-4.11, 9.02); Arrhythmia hospitalizations: 15-64 yrs: -4.04 (-13.3, 6.15), 65+ yrs: 0.10 (-5.91, 6.50); Pneumonia hospitalizations: 15-64 yrs: 0.98 (-8.23, 11.1), 65+ yrs: 0.09 (-5.40, 5.91); Asthma-COPD hospitalizations: 15-64 yrs: 3.23 (-6.84, 14.4), 65+ yrs: 9.62 (2.02, 17.8)
Arbex et al. 2009	Sao Paulo, Brazil	40-64 yrs, 65+ yrs	Short-term; mean 1-hr max 47.88 ppb (SD 22.12 ppb)	COPD-related ED visits		Quantitative results not provided: no association present in any age group
Buadong et al. 2009	Bangkok, Thailand	15-64 yrs, 65+ yrs	Short-term; mean 1-h avg 14.4 ppb	Cardiovascular-related hospitalizations	% change (95% CI) per 5 ppb	15-64 yrs: 0.01 (-0.28, 0.14), 65+ yrs: 0.50 (0.19, 0.81)

Study	Location	Comparison populations	Exposure duration	Health endpoint	Effect estimate	Results
**Sex**						
Cakmak et al. 2006b	Canada	Male, Female	Short-term; mean daily avg 17.4 ppb (SD 21.4 ppb)	Respiratory hospitalizations	% change (95% CI) per 17.4 ppb	Male: 4.5 (2.6, 6.3), Female: 3.6 (1.6, 5.7)
Middleton et al. 2008	Nicosia, Cyprus	Male, Female	Short-term; mean 8-h max ranged by location and season from 28.7 ppb (12.6 ppb) to 54.9 ppb (8.2 ppb)	All-cause hospitalization, respiratory hospitalization, cardiovascular hospitalizations	Percent increase (95% CI)	All-cause hospitalizations: Male: 0.58 (-0.35, 1.52), Female: 0.45 (-0.50, 1.41); Respiratory hospitalizations: Male: -1.76 (-4.63, 1.19), Female: 3.89 (0.12, 7.80); Cardiovascular hospitalizations: Male: 0 (-2.17, 2.22), Female: 1.93 (-1.03, 4.97)
Arbex et al. 2009	Sao Paulo, Brazil	Male, Female	Short-term; mean 1-hr max 47.88 ppb (SD 22.12 ppb)	COPD-related ED visits	% change (95% CI) per 27.93 ppb	Males: quantitative results not provided - no association observed, Females: 2.3 (0.0, 4.5)
Wong et al. 2009	Hong Kong	Male, Female	Short-term; mean 8-h avg 18.45 ppb (SD 11.5 ppb)	Respiratory-related hospitalization, acute respiratory-related hospitalization, COPD-related hospitalization, cardiovascular-related hospitalizations	Excess risk (95% CI) at mean level of influenza activity per 5 ppb	Respiratory-related hospitalizations: Male: 0.84 (0.57, 1.11), Female: 0.87 (0.56, 1.19); Acute respiratory-related hospitalizations: Male: 1.03 (0.63, 1.44), Female: 0.63 (0.18, 1.09); COPD-related hospitalizations: Male: 0.93 (0.51, 1.35), Female: 2.19 (1.63, 2.75); Cardiovascular-related hospitalizations: Male: 0.11 (-0.21, 0.44), Female: 0.25 (-0.07, 0.58)
Paulu and Smith 2008	Maine, U.S.	Male, Female	Short-term; median warm-season 8-hr max ranged by year from 36 ppb to 42 ppb	Asthma-related ED visits	% increase (95% CI) per 10 ppb	Males: 11 (4, 18), Females: 12 (6, 18); By Age and Sex: Males: 2-14 yrs: 17 (3, 32), 15-34 yrs: 10, 35-64 yrs: 11; Females: 2-14 yrs: 4, 15-34 yrs: 20 (10, 31), 35-64 yrs: 8
Thaller et al. 2008	Galveston, TX	Male, Female	Short-term; range of daily avg from 14.62 ppb to 88.69 ppb	FVC, FEV_1/FVC	% change per 10 ppm	FVC: Males: -0.04 (-0.3, 0.4), Females: 0.32 (-0.2, 0.8); FEV_1/FVC: Males: -0.2 (-0.4, 0.03), Females: -0.4 (-0.7, -0.1)

Study	Location	Comparison populations	Exposure duration	Health endpoint	Effect estimate	Results
Lin et al. 2005	Toronto, Canada	Male, Female	Short-term; mean 1-h max 38.06 ppb (17.48 ppb)	Respiratory-infection hospitalizations among children under 15 years old	OR (95% CI) per 21.8 ppb	Males: 1.08 (0.89, 1.31), Females: 1.18 (0.94, 1.47)
Lin et al. 2008	New York State, United States	Male, Female	Short-term; mean 8-h max 41.06 ppb	Asthma-related hospitalizations among children ages 1-6 years		Quantitative results not provided "nonsignificant difference"
Villeneuve et al. 2006	Edmonton, Canada	Male, Female	Short-term; mean daily max 31.2 ppm	Stroke-related ED visits		Quantitative results not provided; no difference apparent in figures with estimates stratified by sex
Henrotin et al. 2007	Dijon, France	Male, Female	Short-term; mean 8-h 14.95 ppb (10.75 ppb)	Ischemic stroke	OR (95% CI) per 5 ppb	Male: 1.058 (0.987, 1.134), Female: 1.036 (0.969, 1.106)
Cakmak et al. 2006a	Canada	Male, Female	Short-term; mean daily avg 17.4 ppb (SD 21.4 ppb)	Cardiac hospitalizations	% change (95% CI) per 17.4 ppb	Male: 1.4 (0.9, 1.9), Female: 2.7 (0.2, 5.2)
Medina-Ramón and Schwartz 2008	U.S.	Male, Female	Short-term; median warm-season 8-hr avg ranged by city from 16.1 ppb to 58.8 ppb	Mortality	Additional % change per 10 ppb	Women compared to men: 0.58 (95% CI 0.18, 0.98); By Age and Sex (compared to men in the same age groups): Women <60yrs: -0.09 (-0.76, 0.58), Women 60+ yrs: 0.60 (0.25, 0.96)
Stafoggia et al. 2010	Italy	Male, Female	Short-term; mean 8-h moving avg ranged by city from 38.95 ppb (SD 10.25 ppb) to 57.7 ppb (SD 19.3 ppb)	Mortality	% increase (95% CI) per 5 ppb	Male: 0.8 (-0.1, 1.8), Female: 2.2 (1.4, 3.1)
Kan et al. 2008	Shanghai, China	Male, Female	Short-term; mean 8-h avg 31.65 ppb (SD 0.5 ppb)	Mortality	% increase (95% CI) per 5 ppb	Male: 0.19 (-0.16, 0.55), Female: 0.40 (0.03, 0.76)
Cakmak et al. 2011	Chile	Male, Female	Short-term; avg 8-h max ozone ranged by city from 59 ppb (SD 56.6 ppb) to 87.6 ppb (86.2 ppb)	Mortality	RR (95% CI) per IQR	Male: 1.052 (1.011, 1.094), Female: 1.042 (1.006, 1.080)

Study	Location	Comparison populations	Exposure duration	Health endpoint	Effect estimate	Results
**Asthma**						
Thaller et al. 2008	Galveston, TX	Asthma, no asthma	Short-term; median daily avg 26 ppb, median daily maximum 35 ppb	FVC, FEV_1/FVC per 10 ppm O_3	% change for daily avg or max O_3	FVC, daily avg O_3: Asthmatic: β -0.4 (95% CI -1.1, 0.4), Non-asthmatic: β 0.09 (95% CI -0.2, 0.4); FVC, daily max O_3: Asthmatic: β -0.07 (95% CI -0.5, -0.4), Non-asthmatic: β 0.09 (95% CI -0.07, 2.5); FEV_1/FVC, daily avg O_3: Asthmatic: β -0.6 (95% CI -1.1, -0.08), Non-asthmatic: β -0.2 (95% CI -0.4, -0.02); FEV_1/FVC, daily max O_3: Asthmatic: β -0.4 (95% CI -0.7, -0.08), Non-asthmatic: β -0.1 (95% CI -0.2, 0)
Escamilla-Nuñez et al. 2008	Mexico City, Mexico	Asthma, no asthma	Short-term; 1-hr daily maximum avg 86.5 ppb (SD 34.4 ppb)	Coughing events, wheezing events per IQR (48.0 ppb) in O_3	% change	Coughing events: Asthmatic: RR 8.9 (95% CI 3.0, 15.1), Non-asthmatic: data not provided “no significant association”; Wheezing events: Asthmatic: RR 10.0 (95% CI 3.2, 17.3), Non-asthmatic: data not provided “no significant association”
Alexeeff et al. 2007	Boston, MA	Airway hyperresponsiveness, no airway hyperresponsiveness	Short-term; mean 2-d avg 24.4 ppb (SD 11.0 ppb)	Lung function (% change in FEV_1 and FVC) per 15 ppb O_3	% change	FEV_1: Nonresponder: OR -1.28 (95% CI -2.01, -0.54), Responder: OR -3.04 (95% CI -4.70, -1.34); FVC: Nonresponder: OR -1.29 (95% CI -1.96, -0.61), Responder: OR -2.03 (95% CI -3.57, -0.46)

Study	Location	Comparison populations	Exposure duration	Health endpoint	Effect estimate	Results
Barraza-Villarreal et al. 2008	Mexico City, Mexico	Asthma, no asthma	Short-term; mean 8-h moving avg 31.6 ppb (SD 11.5 ppb)	FeNO, IL-8, pH_EBC, FEV_1, FVC, FEV_{25-75} per IQR (22.0 ppb) O_3	Linear model, β	FeNO: Asthmatic: 1.06 (95% CI 1.02, 1.09), Non-asthmatic: 1.11 (95% CI 0.92, 1.33); IL-8: Asthmatic: 1.18 (95% CI 1.04, 1.34), Non-asthmatic: 1.19 (95% CI 1.00, 1.45); pH_EBC: Asthmatic: -0.07 (95% CI -0.15, -0.01), Non-asthmatic: -0.07 (95% CI -0.20, 0.05); FEV_1: Asthmatic: -1.64 (95% CI -28.0, 25.1), Non-asthmatic: -21.3 (95% CI -66.5, 23.9); FVC: Asthmatic: -13.5 (95% CI -45.0, 19.0), Non-asthmatic: -23.6 (95% CI -75.0, 28.1); FEV_{25-75}: Asthmatic: 24.3 (95% CI -29.0, 78.2), Non-asthmatic: -14.5 (95% CI -118.7, 89.5)
Berhane et al. 2011	Southern CA	Asthma, no asthma; respiratory allergy, no respiratory allergy	Short-term; 8-hr daily avg, IQR 15.42 ppb	FeNO change per IQR (15.42 ppb) O_3	% change	Asthmatic: β 12.6 (95% CI -0.7, 27.7), Non-asthmatic: β 14.5 (95% CI 5.3, 24.4); Respiratory allergy: β 15.4 (95% CI 6.0, 25.7), No respiratory allergy: β 12.4 (95% CI 2.7, 23.0)
**Obesity**						
Alexeeff et al. 2007	Boston, MA	BMI <30 kg/m^2, BMI ≥30 kg/m^2	Short-term; mean 2-d avg 24.4 ppb (SD 11.0 ppb)	Lung function (% change in FEV_1 and FVC) per 15 ppb O_3	% change	FEV_1: BMI <30 kg/m^2: OR -1.15 (95% CI -1.91, -0.39), BMI ≥30 kg/m^2: OR -2.63 (95% CI -3.85, -1.39); FVC: BMI <30 kg/m^2: OR -1.18 (95% CI -1.88, -0.48), BMI ≥30 kg/m^2: OR -2.05 (95% CI -3.17, -0.91)

Study	Location	Comparison populations	Exposure duration	Health endpoint	Effect estimate	Results
**Diet**						
Romieu et al. 2009	Mexico City, Mexico	Fruit and vegetable index, Mediterranean diet index	Short-term; mean 8-h moving avg 31.6 ppb	IL-8, FEV_1, FVC	Coefficient (SE) for the relationship between increasing dietary index and outcomes by quartile of ozone exposure	Fruit and vegetable index: IL-8: ≤25 ppb: -0.125 (0.094), ≥38 ppb: -0.219 (0.084), FEV_1: ≤25 ppb: 0.049 (0.061), ≥38 ppb: 0.099 (0.058), FVC: ≤25 ppb: 0.065 (0.069), ≥38 ppb: 0.137 (0.066); Mediterranean diet index: IL-8: ≤25 ppb: -0.020 (0.055), ≥38 ppb: -0.022 (0.048), FEV_1: ≤25 ppb: 0.048 (0.033), ≥38 ppb: 0.051 (0.032), FVC: ≤25 ppb: 0.048 (0.037), ≥38 ppb: 0.081 (0.036)

Study	Location	Comparison populations	Exposure duration	Health endpoint	Effect estimate	Results
Sienra-Monge et al. 2004	Mexico	Vitamin supplementation (50 mg/day of vitamin E and 250 mg/day of vitamin C), Placebo	Short-term; mean ozone 8-h moving avg 66.2 ppb	Inflammation per 100 ppb O_3 (interleukin-6 (IL-6), interleukin-8 (IL-8), glutathione (GSx), uric acid	β	IL-6, 1-day lag: Placebo 0.98 (SE 0.44; p-value 0.02), Supplement -0.02 (SE 0.42; p-value 0.97); IL-6, 3-day lag: Placebo 1.07 (SE 0.30; p-value <0.01), Supplement -0.04 (SE 0.31; p-value 0.90); IL-6, 3-day cumulative: Placebo 1.44 (SE 0.47; p-value <0.01), Supplement 0.09 (SE 0.52; p-value 0.86); IL-8, 1-day lag: Placebo 0.80 (SE 0.49; p-value 0.10), Supplement -0.26 (SE 0.49; p-value 0.60); IL-8, 3-day lag: Placebo 0.78 (SE 0.37; p-value 0.04), Supplement 0.29 (SE 0.38; p-value 0.45); IL-8, 3-day cumulative: Placebo 1.15 (SE 0.55; p-value 0.04), Supplement 0.02 (SE 0.62; p-value 0.98); GSx, 2-day lag: Placebo -0.13 (SE 0.15; p-value 0.39), Supplement -0.29 (SE 0.13; p-value 0.03); GSx, 3-day lag: Placebo -0.27 (SE 0.10; p-value <0.01), Supplement -0.06 (SE 0.09; p-value 0.51); GSx, 3-day cumulative: Placebo -0.35 (SE 0.16; p-value 0.03), Supplement -0.33 (SE 0.14; p-value 0.02); Uric acid, 2-day lag: Placebo -0.25 (SE 0.38; p-value 0.51), Supplement 0.08 (SE 0.41; p-value 0.85); Uric acid, 3-day lag: Placebo -0.34 (SE 0.27; p-value 0.20), Supplement 0.41 (SE 0.28; p-value 0.14); Uric acid, 3-day cumulative: Placebo -0.42 (SE 0.40; p-value 0.30), Supplement 0.34 (SE 0.46; p-value 0.46)

Study	Location	Comparison populations	Exposure duration	Health endpoint	Effect estimate	Results
**SES**						
Cakmak et al. 2006b	Canada	Neighborhood-level education (< grade 9, grades 9-13, some university or trade school, university diploma) and neighborhood-level income (<$21,309, $21,309-$28,161, $28,161-$35,905, >$35,905)	Short-term; mean daily avg 17.4 ppb (SD 21.4 ppb)	Respiratory hospitalizations	% change (95% CI) per 17.4 ppb	Neighborhood-level education: < grade 9: 4.0 (1.6, 6.5), grades 9-13: 1.4 (-1.6, 4.4), some university or trade school: 1.1 (-1.6, 4.0), university diploma: 0.5 (-2.5, 3.5); Neighborhood-level income: <$21,309: 2.8 (0.4, 5.1), $21,309-$28,161: 5.6 (3.0, 8.2), $28,161-$35,905: 2.9 (0.1, 5.7), >$35,905: 4.6 (1.8, 7.5)
Lee et al. 2006	Seoul, Korea	Neighborhood level insurance rate (lower, intermediate, upper)	Short-term; mean 8-h max avg 29.83 ppb (SD 16.87 ppb)	Asthma-related hospitalizations for children <15 yrs	RR (95% CI) per IQR (ranged 23.94 ppb to 33 ppb by grp)	Lower: 1.32 (1.11, 1.58), Intermediate: 1.24 (1.08, 1.43), Upper: 1.12 (1.00, 1.25)
Burra et al. 2009	Toronto, Canada	Quintiles of neighborhood average household income (quintile 1: $18,901-$42,688; quintile 5: $76,032-$245,701)	Short-term; avg daily 1-h max ozone 33.3 ppb (SD 16.4 ppb)	Ambulatory physician visits for asthma among children aged 1-17 yrs	RR (95% CI)	Quintile 1: Males 0.961 (95% CI 0.956, 0.966), Females 0.955 (95% CI 0.949, 0.961); Quintile 5: Males 0.966 (95% CI 0.961, 0.972), Females 0.962 (95% CI 0.955, 0.969)
Cakmak et al. 2006a	Canada	Neighborhood-level education (quartiles) and neighborhood-level income (quartiles: <$21,309, $21,309-$28,161, $28,161-$35,905, >$35,905)	Short-term; mean daily avg 17.4 ppb (SD 21.4 ppb)	Cardiac hospitalizations	% change (95% CI) per 17.4 ppb	Neighborhood-level education: First quartile: 1.4 (-0.9, 3.7), Second quartile: -2.8 (-8.6, 3.0), Third quartile: 6.0 (0.2, -11.8), Fourth quartile: 2.2 (-3.8, 8.2); Neighborhood-level income: <$21,309: 1.8 (-0.7, 4.3), $21,309-$28,161: 1.0 (-2.0, 4.0), $28,161-$35,905: 3.0 (0.6, 5.4), >$35,905: 1.3 (-1.7, 4.3)
Bell and Dominici 2008	U.S.	Unemployment, poverty, median income	Short-term; mean daily ozone 26.8 ppb (IQR 6.4 ppb)	Mortality	% change in effect estimate (95% PI) for O_3 and mortality per IQR increase in neighborhood variable	Unemployment: 68.3 (3.02, 133.7), Poverty: 35.4 (-24.7, 95.4), Median income: 9.34 (-42.5, 61.2); Adjusted for neighborhood variable: Unemployment: 0.49 (0.26, 0.73), Poverty: 0.51 (0.27, 0.75), Median income: 0.52 (0.28, 0.77)

Study	Location	Comparison populations	Exposure duration	Health endpoint	Effect estimate	Results
Katsouyanni et al. 2009	U.S., Canada, Europe	Unemployment	Short-term; mean 1-h max ranged from 13.3-38.4 ppb in the US, 6.7-8.4 ppb in Canada, and 18.3-41.9 ppb in Europe	Mortality	% change (95% CI) per 5 ppb	United States: 25%ile of unemployment: 0.02 (-0.10, 0.15), 75%ile of unemployment: 0.19 (0.09, 0.29); Canada: 25%ile of unemployment: 0.35 (0.18, 0.51), 75%ile of unemployment: 0.47 (0.32, 0.61); Europe: 25%ile of unemployment: 0.18 (-0.06, 0.42), 75%ile of unemployment:: 0.17 (-0.06, 0.40)
Cakmak et al. 2011	Chile	Individual education (primary school not completed, primary school completed, high school completed, some college, college completed), Neighborhood income quartile (<$8800, $8800-$10,651, $10, 651-$13,395, >$13,395), Employment category (unemployed, blue-collar, white-collar)	Short-term; avg 8-h max ozone ranged by city from 59 ppb (SD 56.6 ppb) to 87.6 ppb (86.2 ppb)	Mortality	RR (95% CI) per IQR	Individual education: Primary school not completed: 1.094 (1.039, 1.140), Primary school completed: 1.061 (1.016, 1.106), High school completed: 1.050 (1.007, 1.092), Some college: 1.037 (0.995, 1.080), College completed: 1.034 (0.991, 1.078); Neighborhood income quartile: <$8800: 1.081 (1.049, 1.113), $8800-$10,651:1.062 (1.027, 1.097), $10, 651-$13,395: 1.030 (1.000, 1.060), >$13,395: 1.023 (0.994, 1.052); Employment category: Unemployed: 1.089 (1.032, 1.146), Blue-collar: 1.028 (0.994, 1.061), White-collar: 0.980 (0.931, 1.029)
Kan et al. 2008	Shanghai, China	Individual low education (illiterate or primary school, high education (middle school or more)	Short-term; mean 8-h avg 31.65 ppb (SD 0.5 ppb)	Total mortality, cardiovascular mortality, respiratory mortality	% increase (95% CI) per 5 ppb	Total mortality: Low education: 0.26 (-0.09, 0.60), High education: 0.30 (-0.11, 0.71); Cardiovascular mortality: Low education: 0.39 (-0.13, 0.90), High education: 0.26 (-0.38, 0.91); Respiratory mortality: Low education: 0.20 (-0.74, 1.16), High education: 0.27 (-0.86, 1.41)

Study	Location	Comparison populations	Exposure duration	Health endpoint	Effect estimate	Results
Wong et al. 2008	Hong Kong, China	Neighborhood social deprivation index, based on unemployment, household income, education, number of people in the household, marital status, and subtenancy	Short-term; mean 8-h avg 18.45 ppb (SD 11.5 ppb)	Total mortality, cardiovascular mortality, respiratory mortality	% increase (95% CI) per 5 ppb	Total mortality: Low deprivation: 0.22 (-0.26, 0.70), Middle deprivation: 0.46 (0.09, 0.83), High deprivation: 0.02 (-0.49, 0.54); Cardiovascular mortality: Low deprivation: 0.41 (-0.53, 1.35), Middle deprivation: 0.65 (-0.04, 1.34), High deprivation: 0.23 (-0.71, 1.18); Respiratory mortality: Low deprivation: 0.46 (-0.68, 1.61), Middle deprivation: 0.26 (-0.56, 1.09), High deprivation: -0.51 (-1.65, 0.64)
Stafoggia et al. 2010	Italy	Neighborhood-level low income, medium income, high income	Short-term; mean 8-h moving avg ranged by city from 38.95 ppb (SD 10.25 ppb) to 57.7 ppb (SD 19.3 ppb)	Mortality	% increase (95% CI) per 5 ppb	Neighborhood-level low income: 1.9 (0.5, 3.4), Neighborhood-level medium income: 1.7 (0.8, 2.6), Neighborhood-level high income: 2.2 (0.7, 3.9)
Romieu et al. 2004	Ciudad Juarez, Mexico	High SES, Medium SES, Low SES; SES measured by social deprivation indexes (based on available public services)	Short-term; annual avg of daily value ranged from 43.43 ppb (SD 19.57 ppb) to 55.12 ppb (SD 20.72 ppb)	Infant mortality		Quantitative results not given
Carbajal-Arroyo et al. 2011	Mexico City, Mexico	Area-level neighborhood socioeconomic index, based on variables related to education, income, and housing condition	Short-term; mean 1-h max 103.0 ppb (SD 34.2 ppb)	Infant mortality (all-cause and respiratory)	OR (95% CI) per 69 ppb	All-cause: Low SES: 1.076 (0.980, 1.182), Medium SES: 1.041 (0.973, 1.113), High SES: 0.932 (0.875, 0.993); Respiratory: Low SES: 1.264 (1.079, 1.481), Medium SES: 0.997 (0.889, 1.119), High SES: 0.983 (0.872, 1.108)
Morello-Frosch et al. (2010)	California	Neighborhood-level poverty rate: 0-7%, 7-14%, 14-22%, 22-32%, ≥32%	Long-term; daily mean avged over pregnancy 23.5 (SD 6.5 ppb)	Birthweight		Quantitative results not given; in figure lower birth weights in association with change in ozone were observed in all grps except 0-7%. The change was not linear.

Study	Location	Comparison populations	Exposure duration	Health endpoint	Effect estimate	Results
Hansen et al. 2008	Australia	Neighborhood socioeconomic index (quartiles), based on income, education, unemployment, and occupations	Long-term; mean 8-hr avg 24.8 ppb (IQR 9.8 ppb)	Fetal ultrasonic abdominal circumference	Mean change per 9.8 ppb	Quartile 1: -2.18 (-5.39, 1.02), Quartile 2: -1.33 (-3.17, 0.50), Quartile 3: -0.94 (-2.33, 0.45), Quartile 4: -1.50 (-2.78, -0.22); Note: change for other fetal measurements (femur length, biparietal diameter, and head circumference) not given because none of the overall associations with ozone were "statistically significant;" therefore, modification was not examined
**Outdoor workers**						
Tovalin et al. 2006	Mexico (Mexico City and Puebla)	Outdoor workers, indoor workers	Short-term; Mexico City: medians of: 28.5 ppb and 5.1 ppb for outdoor and indoor workers, respectively, Puebla: medians of 36.1 ppb and 19.5 ppb, for outdoor and indoor workers, respectively	DNA damage (Comet assay tail length)		Mexico City: Correlation for outdoor workers: R=0.42, p (0.06), No correlation for indoor workers (quantitative results not provided); Puebla: No correlation for indoor or outdoor workers (quantitative results not provided)

Table S4. Results from controlled human exposure studies examining response modifying factors for O_3-related health effects (listed in order of appearance within each section).

Study	Location	Population/animal model	Exposure (O_3 concentration [ppm], duration)	Health endpoint	Results
**Lifestage**					
McDonnell et al. 1999	NC, USA	Adults, M, 18-35 years, healthy	0.0, 0.12, 0.18, 0.24, 0.30, or 0.40 ppm, 2 h	Respiratory symptoms, FEV_1	Maximum response (i.e., FEV_1 and symptoms, except cough) is inversely related to age
**Sex**					
Bush et al. 1996	PA, USA	Adults, M/F, 22-35 years old	20 mL bolus of 3 ppm	Bolus penetration, respiratory anatomy (FVC, V_D, TLC)	O_3 penetration was deeper in women than men; however, when normalized to anatomical dead space no difference was observed.
Hazucha et al. 2003	NC, USA	Adults, M/F, 18-60 years old	0.42 ppm, 1.5 h	FEV_1	No significant difference between males and females
**Asthma**					
Horstman et al. 1995	NC, USA	Adults, M/F, 18-35 years old, asthmatics and nonasthmatics	0.16 ppm, 7.6 h	FEV_1, FVC, respiratory symptoms, bronchodilator use	Asthmatics had greater decrements in FEV_1 and FEV_1/FVC but not FVC compared to nonasthmatics. Asthmatics had greater wheezing than nonasthmatics.
Kreit et al. 1989	MI, USA	Adults, M/F, 18-34 years old, asthmatics and nonasthmatics	0.4 ppm, 2 h	FEV_1, FVC, FEF_{25-75}, FEV_1%, sRaw, respiratory symptoms	Greater percent decrease in FEV_1, FEV_1%, and FEF_{25-75} in asthmatics. No difference between asthmatics and nonasthmatics on FVC and symptoms.
Alexis et al. 2000	Canada	Adults, M/F, 18-34 years old, asthmatics and nonasthmatics	0.4 ppm, 2 h	FEV_1, FVC, FEF_{25}, FEF_{50}, FEF_{75}, prostaglandin F2-α	Similar decrements in FVC and FEV_1 in asthmatics and nonasthmatics. Asthmatics had greater decrement in FEF_{75} (small airway function). Indomethacin attenuated O_3-induced decrements in FVC, FEV_1 in nonasthmatics but not asthmatics and FEF60p and FEF_{75} in asthmatics but not nonasthmatics. Higher PGF2-α levels in asthmatics post O_3 exposure.
Jorres et al. 1996	Germany	Adults, M/F, allergic asthmatics, allergic rhinitis without asthma, and nonasthmatics	0.25 ppm, 3 h	FEV_1, methacholine and allergen responsiveness	Mean O_3-induced FEV_1 decrements not different between asthma, rhinitis, and healthy. Methacholine and allergen responsiveness increased in asthmatics, smaller shift in rhinitis, no change in healthy.

Study	Location	Population/animal model	Exposure (O_3 concentration [ppm], duration)	Health endpoint	Results
Mudway et al. 2001	Sweden	Adults, M/F, median 25 years old, mild atopic asthmatics and healthy	0.2 ppm, 2 h	Respiratory symptoms, FEV_1, FVC, bronchial wash and BALF protein, neutrophils, albumin, and antioxidants,	No change in the O_3-induced FEV_1 decrement and BALF or BW neutrophils, albumin or protein between healthy and asthmatic subjects. O_3 decreased BW and BALF ascorbate and increased GSSG in healthy subjects but not asthmatics, which had lower and higher baseline levels, respectively. O_3 increased EC-SOD in healthy subjects. No O_3 effect on MDA, urate, α-tochopherol.
Basha et al. 1994	MI, USA	Adults, M, 18-45 years old, healthy and asthmatic	0.2 ppm, 6 h	Respiratory symptoms, sRaw, FVC, IC, FEV_1, FEF, BALF albumin, cytokines, cell count	No difference in lung function between groups. Increased PMNs, IL-6, and IL-8 in asthmatics compared to healthy.
Peden et al. 1997	NC, USA	Adults, M, mild asthmatics	0.16 ppm, 7.6 h	BALF eosinophils, lung function (FEV_1, FVC)	Increased eosinophils and PMN's after O_3 exposure more in initial (bronchial) fraction. No correlation of eosinophils and PMN's, FEV_1 and FVC decreased 14% and 9%, respectively.
Scannell et al. 1996	CA, USA	Adults, M/F, 26 ± 5.4 years old, mild asthmatics	0.2 ppm, 4 h	Lung function (FEV_1, FVC), BALF cell counts	FVC, FEV_1 decreased 17.6% and 25% respectively. Spirometry changes in asthmatics similar to healthy subjects (Aris et al., 1995; Balmes et al., 1997). Trend for larger increase in SRaw in asthmatics. Larger increase in PMN's and protein in asthmatics indicating more inflammation. No increase in eosinophils.
Hernandez et al. 2010	NC, USA	Adults, M/F, 19-39 years old, healthy, atopic, and atopic asthmatics	0.4 ppm, 2 h	Lung function, sputum cytokines, leukocyte cell-surface molecules, and hyaluronic acid	Similar decrements in lung function in all groups. Increased sputum neutrophil and IL-8 and HA in asthmatics and atopics. Asthmatics had increased sputum IL-6 and IL-1β and airway TLR4, CD23, FcεRI.
Bosson et al. 2003	Sweden	Adults, M/F, 19-48 years old, healthy and mild asthmatic	0.2 ppm, 2 h	Endobronchial mucosal biopsy, cell markers, cytokines, and adhesion molecules expression	Significantly higher baseline expression of IL-4 and IL-5 in bronchial mucosal biopsies from asthmatic vs. healthy subjects 6 h postexposure. Following O_3 exposure, epithelial expression of IL-5, GM-CSF, ENA-78, and IL-8 increased significantly in asthmatics, as compared to healthy subjects.

Study	Location	Population/animal model	Exposure (O_3 concentration [ppm], duration)	Health endpoint	Results
**Obesity**					
McDonnell et al. 2010	NC, USA	Adults, M/F, age 18-35 years, data from 15 controlled human exposure studies conducted at US EPA facility	0.08-0.4 ppm, 1-7.6 h, from 1981-1992	Model predicted FEV_1 response	Increasing BMI associated with increasing magnitude of FEV_1 response to ozone (estimated BMI coefficient statistically signif different from zero, 0.4855 [0.1018, 0.8693])
Bennett et al. 2007	NC, USA	Adults, M/F, age 18-35 years,	0.42 ppm, 1.5 h, with IE	Spirometry (FEV_1, FVC, FEF_{25-75})	Change in FEV_1 after ozone was inversely correlated with BMI (r =-0.016). More obviously in females, not statistically signif in males. Similar for change in FEF_{25-75}.
**Diet**					
Samet et al. 2001	NC, USA	Adults, 18-35 years (mean 26 years), M/F	0.4 ppm, 2 h, following 2 week antioxidant supplementation or not (vitamin C, vitamin E, vegetable cocktail)	Pulmonary function, BALF cellularity and cytokines	Antioxidant supplementation blunted the O_3-induced decrement in and FVC. No change in O_3-induced respiratory symptoms or BALF PMN and BALF IL-6 increases.
Trenga et al. 2001	WA, USA	Adults with asthma, mean 27 years, M/F	0.12 ppm, 45 min, challenged with 0.1 and 0.25 ppm SO_2 for 10 min, supplemented or not with vitamins (vitamins E and C) for 4 weeks prior	SO_2 induced bronchial hyperresponsiveness, pulmonary function,	Antioxidant supplementation led to less severe responses to SO_2 challenge. FEV_1: -1.2% vs -4.3%; peak flow: 2.2% vs -3.0%; mid-forced expiratory flow 2% vs -4.3%
**SES**					
Seal et al. 1996	NC, USA	Adults, M/F, 18-35 years old (mean 23.9 ±4.4 years)	0, 0.12, 0.18, 0.24, 0.3, or 0.4 ppm, 2.3 h	Pulmonary function (FEV_1)	Individuals from a medium SES (education of father) most responsive to O_3 induced FEV_1 decrements, followed by low and high SES.

Study	Location	Population/animal model	Exposure (O_3 concentration [ppm], duration)	Health endpoint	Results
**Outdoor Workers**					
Hu et al. 1994	PA, USA	Adults, M, mean 32.3±6.7 years old	10 ml bolus of 3.0 ppm O_3 at respiratory flows of 150, 250, 500, 750, and 1000 ml/s	Absorbed fraction (amount of O_3 absorbed during a single respiratory cycle relative to inhaled amount), penetration volume (mean airway volume that would be reached by O_3 molecules during inhalation if no absorption occurred), Vb (breakthrough volume, mean airway volume traversed by unabsorbed O_3 molecules during expiration), dispersion (measure of longitudinal mixing of unabsorbed O_3 molecules)	At a fixed flow, absorbed fraction increased with penetration volume and elevating flow rate shifted the absorbed fraction distribution distally into the lungs so a greater amount of O_3 reached the small airways and air spaces and less O_3 was absorbed in the upper airways and conducting airways.
Nodelman and Ultman 1999	PA, USA	Adults, M/F, mean 24.7 ± 5.4 years old	20 ml bolus of 1.0 ppm O_3 at respiratory flows of 150, 250, and 1000 ml/s	Absorbed fraction (amount of O_3 absorbed during a single respiratory cycle relative to inhaled amount), penetration volume (mean airway volume that would be reached by O_3 molecules during inhalation if no absorption occurred)	Larger penetrations of O_3 beyond the upper airways occurred as flow increased and during nasal than during oral breathing

Table S5. Results from toxicological studies examining response modifying factors for O_3-related health effects (listed in order of appearance within each section).

Study	Location	Population/animal model	Exposure (O_3 concentration [ppm], duration)	Health endpoint	Results
**Lifestage**					
Fanucchi et al. 2006	CA, USA	Monkey (*Macaca mulatta*), M, 30 days	0.5 ppm, 8 h/day for 5 days followed by 9 days of filtered air, 5 months of episodic exposure	Lung morphology and structure, lung function	Fewer nonalveolarized airway generations, reduction of distal airway size, hyperplastic bronchiolar epithelium, altered smooth muscle bundle orientation in terminal and respiratory bronchioles
Carey et al. 2007	CA, USA	Monkey (*Macaca mulatta*), M, 30 days	0.5 ppm, 8 h/day for 5 days followed by 9 days of filtered air, 5 days or 70 days of episodic exposure	Nasal airway histopathology, imaging, and modeling	Neutrophilic rhinitis, necrosis and exfoliation of epithelium lining of the anterior maxilloturbinate
Harkema et al. 1987	CA, USA	Monkey (*Macaca radiata*), M/F, 2-6 years	0.15 or 0.3 ppm, 8 h/day for 6 or 90 days	Nasal airway histopathology	Ciliated cell necrosis, shortened cilia, secretory cell hyperplasia, ultrastructural goblet cell changes (90 days), inflammatory cell influx (6 days)
Plopper et al. 2007	CA, USA	Monkey (*Macaca mulatta*), M, 30 days	0.5 ppm, 8 h/day for 5 days followed by 9 days of filtered air, 5 months of episodic exposure	Lung morphology and structure, lung function, immunologic responses	Increased BALF eosinophils, decreased number of conducting airway generations, reduced airway diameter and length growth, bronchial epithelium hyperplasia, reorganization of the airway vascular and immune system, modified epithelial nerve fiber distribution, altered distal airway smooth muscle bundle orientation and abundance, increase in mucous goblet cells
López et al. 2008	Mexico	Rats (Wistar), F, pregnant	1 ppm, 12 h/day, 18, 20, and 21 days of gestation	Ultrastructural analysis	GD18: swollen mitochondria, cyoplasmic vacuolization, structural architecture disarrangement, cytoplasm rupture GD20: flake-off epithelial cells and lumenal laminar bodies GD21: edematous mitochondria, mitochondria cristae damage
Auten et al. 2009	NC, USA	Mice (C57BL6), dams and litters	1 ppm, 3 h/day, every other day, 3 times a week, 4 weeks	Airway responsiveness, inflammatory markers, lung morphometry	Increased maternal whole lung cytokines (IL-1β, TNFα, IL-6, KC, MCP-1), increased airway hyperresponsiveness (total and large airway resistance)

Study	Location	Population/animal model	Exposure (O_3 concentration [ppm], duration)	Health endpoint	Results
Vancza et al. 2009	NY, USA	Mice (A/J, AKR/K, C3H/HeJ, BALB/cJ, C57BL/6J, DBA/J, SJL/J, 129x1/SvJ), neonatal (15-16 days old), lactating and non-lactating female (15 weeks old), adult male (15 weeks old)	0.8 ppm, 5 h	Lung inflammation (PMN) and injury (protein) (BALF) 24-h postexposure	Neonates had increased protein compared to adults in two strains. Adults had increased protein compared to neonates in three strains. Neonates had increased PMN compared to adults in two strains. Adults had increased PMNs in one strain. Significant interaction between age and total PMNs and strain:exposure: age for total protein and total PMNs.
Bils 1970	CA, USA	Mice (IVAN-NMRI), 4 days, 1 month, 2 months old	0.6-1.3 ppm, 6-7 h/day, 1-2 days	Lung morphology	Youngest mice were most sensitive to alveolar damage with endothelial cells lining the capillaries as a main target.
Servais et al. 2005	France	Rats (Sprague-Dawley), M, 3 weeks (young), 6 months (adult), and 20 months (aged) old	0.5 ppm, 12 h/day, 7 days	Ventilation, pulmonary mitochondria respiration and H2O2 release, pulmonary antioxidant enzyme activity, DNA 8-oxodG and HNE-dG content, HSP72 content	Young rats had higher nDNA 8-oxodG and HSP72 content than adult rats. Aged rats had mild uncoupled lung mitochondria, increased SOD and GPx activities and higher 8-oxodG content than adult rats.
Fortino et al. 2007	Italy	Mice (SKH-1 hairless mice), F, 8 weeks and 18 months old	0.25 ppm, 6 h/day, 4 days	Cutaneous MMP protein and activity	Increased MMP-2 in young but not old mice; no change in MMP-9. Increased MMP-12 in older mice.
Hamade and Tankersley 2009	MD, USA	Mice (C57Bl/6J, C3H/HeJ, and C3H/HeOuJ), M, 18-20 weeks	0.6 ppm, 2 h/day, 3 days, followed by 536 $\mu g/m^3$ carbon black, 3 h	Heart rate, heart rate variability, respiratory responses (respiratory rate, tidal volume, ventilation), core temperature	Strains varied in integration of the cardiac and respiratory systems, implications in interindividual variability. B6 mice were mildly responsive with rapid adaptation, whereas C3 mice were highly responsive with adaptation only in HeJ mice with regards to changes in cardiac and respiratory responses.
Tankersley et al. 2010	MD, USA	Mice (C57Bl/6J, 129S1/SvlmJ), M/F, 5 or 18 months	0.6 ppm, 2 h, followed by 556 $\mu g/m^3$ carbon black, 3 h	Echocardiographic and in vivo hemodynamic measurements (e.g., HR; PWTED, posterior wall thickness at end-diastole; LVESD, left ventricular diameter at end systole)	Significant interaction between age and strain on HR and PWTES, which implies that aging affects the HR and function in response to O_3 differently between mouse strains.

Study	Location	Population/animal model	Exposure (O_3 concentration [ppm], duration)	Health endpoint	Results
Hamade et al. 2010	MD, USA	Mice (C57Bl/6J, C3H/HeJ, and C3H/HeOuJ), M, 5 or 12 months	0.6 ppm, 2 h, followed by 536 μg/m^3 carbon black, 3 h	Heart rate, heart rate variability, breathing, core temperature	Aged mice exhibited attenuated changes in cardiopulmonary physiology after O_3 exposure. Genetic differences between mice strains could be altering formation of ROS, which tends to increase with age, thus modulating O_3 induced effects.
Lim et al. 2006	CA, USA	Mice (SKH-1 hairless mice), F, 8 weeks and 18 months old	0.5 ppm, 6 h/day, 9 days	Wound closure, lipid peroxidation, protein oxidation, transcription factor levels	Older mice had delayed wound closer, increased 4-HNE protein adducts and protein carbonyls, decreased p-IκBα and TGFβ protein.
Rivas-Arancibia et al. 2000	Mexico	Rats (Wistar), M, 47 days (young), 540 days (adult), 900 days (aged) old	0.7-1.0 ppm, 4 h	Motor activity, one-trial passive avoidance conditioning, lipid peroxidation	Young and aged rats had decreased short-term and long-term memory, not adult rats. Lipid peroxidation increased in young and aged rats in the striatum, hippocampus, and frontal cortex. Lipid peroxidation increased in young rats cerebellum.
**Sex**					
Vancza et al. 2009	NY, USA	Mice (A/J, AKR/K, C3H/HeJ, BALB/cJ, C57BL/6J, DBA/J, SJL/J, 129x1/SvJ), neonatal (15-16 days old), lactating and nonlactating female (15 weeks old), adult male (15 weeks old)	0.8 ppm, 5 h	Lung inflammation and injury (BALF) 24-h postexposure	Nonlactating females had larger increases in protein compared to males in most strains (6 of 8) and largest increase in PMN in one strain. Lactating females had the largest increase in protein in 6 of 8 strains (2 statistically signif) and largest increase in PMN in 3 of 8 (1 statist signif). Signif interactions between exposure:sex for protein.
**Asthma**					
Fanucchi et al. 2006	CA, USA	Monkey (*Macaca mulatta*), M, 30 days	0.5 ppm, 8 h/day for 5 days followed by 9 days of filtered air, 5 months of episodic exposure	Lung morphology and structure, lung function	Fewer nonalveolarized airway generations, reduction of distal airway size, hyperplastic bronchiolar epithelium, altered smooth muscle bundle orientation in terminal and respiratory bronchioles

Study	Location	Population/animal model	Exposure (O_3 concentration [ppm], duration)	Health endpoint	Results
Joad et al. 2006	CA, USA	Monkey (*Macaca mulatta*), M, 30 days	0.5 ppm and 0.5 ppm+house dust mite allergen (2 h/day, last 3 days of ozone), 6 h/day for 5 days followed by 9 days of filtered air, 5 months of episodic exposure	Methacholine airway responsiveness, lung histology	Ozone+HDMA increased respiratory bronchiole airway responsiveness compared to FA, ozone, or allergen alone.
Schelegle et al. 2003	CA, USA	Monkey (*Macaca mulatta*), M, 30 days	0.5 ppm and 0.5 ppm+house dust mite allergen (2 h/day, last 3 days of ozone), 8 h/day for 5 days followed by 9 days of filtered air, 5 months of episodic exposure	Pulmonary mechanics, airway responsiveness, plasma histamine, BALF cell counts, serum IgE, histopathology	Ozone+HDMA increased serum IgE, serum histamine, baseline airway resistance (R_{aw}), airway responsiveness ($EC150R_{aw}$), and airways eosinophilia. Also, combined exposure resulted in greater alterations in airway structure and content than ozone or HDMA alone.
Funabashi et al. 2004	Japan	Mice (C57BL/6), M	1.0 ppm with or without exposure to ovalbumin (30 min, 3 times), 6 h/day, 5 days/week for 5 weeks	Pulmonary function	With OA sensitization, O_3 increased respiratory resistance and decreased dynamic compliance
Funabashi et al. 2004	Japan	Mice (C57BL/6), M	1.0 ppm, 1 h following repeated exposure	Pulmonary function, arterial blood gas, histopathology	No change in PaO_2 after O_3, alveolar epithelial hyperplasia after O_3, perivascular infiltration of eosinophils and histiocytes with alveolar epithelial hyperplasia after O_3+OA
Wagner et al. 2007	MI, USA	Rat (Brown Norway), M	1.0 ppm with or without exposure to ovalbumin (days 1 and 2), 8 h/day, days 4 and 5, and γ-tocopherol on days 2-5	BALF cellularity, cytokine content, leukotriene content, tocopherols, intraepithelial mucosubstances morphometry, tissue eosinophil density	Ozone exposure of allergic rats enhanced intraepithelial mucosubstances increases in proximal axial airways (200%), induced cys-leukotrienes, MCP-1, and IL-6 production in BALF, and upregulated expression of IL-5 and IL-13 mRNA. Attenuated by γ-T treatment.

Study	Location	Population/animal model	Exposure (O_3 concentration [ppm], duration)	Health endpoint	Results
Oyarzún et al. 2005	Chile	Rat (Sprague Dawley)	0.25 ppm, 4 h/day, 5 days/week, for 5 and 60 days, 30 days after bleomycin instillation	Histopathology	No change in lung damage after 60 days compared to bleomycin alone. Increased mean score of pulmonary inflammation and fibrosis after 5 days of ozone. Increased frequency of bronchopneumonia and detachment of bronchiolar epithelium after 5 days of ozone.
**Obesity**					
Johnston et al. 2008	MA, USA	Mice (C57BL/6J, diet-induced obesity *[C57BL/6J* 60% calories from fat vs. 10%]*), M/F*	2 ppm, 3 h	Pulmonary mechanics, BALF cellularity and cytokines	Obesity increased O_3-induced BALF protein (hyperpermeability marker), IL-6, Eotaxin, KC, MIP-2, IP-10, sTNFR1, sTNFR2, over lean O_3 exposure.
Shore et al. 2009	MA, USA	Mice (C57BL/6J, *Db/db*, *Cpe^{fat}*, diet-induced obesity *[C57/IL6$^{-/-}$* 60% calories from fat vs. 10%]*), F (Db/db) M/F (Cpe^{fat}, C57BL/6J)*	0.3 ppm, 12-72 h	Pulmonary mechanics, BALF cellularity and cytokines, serum cytokines	Obesity (genetic and diet induced) blunted the subacute (72 h) O_3-induced decrease in Cdyn, increase in BALF macrophages and neutrophils, increase in BALF protein, and increase in BALF sTNFR1. IL-6 deficiency attenuated O_3 induced increase in BALF macrophages and neutrophils. Obesity related difference in O_3 inducted neutrophil influx was dependent on IL-6. Discordance with acute exposure, obesity increased acute (12-48 h) O_3 induced increased BALF protein, BALF macrophages, and BALF sTNFR1.
**Diet**					
Wagner et al. 2007	MI, USA	Rat (Brown Norway), M	1.0 ppm with or without exposure to ovalbumin (days 1 and 2), 8 h/day, days 4 and 5, and γ-tocopherol on days 2-5	BALF cellularity, cytokine content, leukotriene content, tocopherols, intraepithelial mucosubstances morphometry, tissue eosinophil density	Ozone exposure of allergic rats enhanced intraepithelial mucosubstances increases in proximal axial airways (200%), induced cys-leukotrienes, MCP-1, and IL-6 production in BALF, and upregulated expression of IL-5 and IL-13 mRNA. Attenuated by γ-T treatment.
Wagner et al. 2009	MI, USA	Rat (Brown Norway), M	1.0 ppm with or without exposure to ovalbumin (days 1 and 2), 8 h/day, days 4 and 5, and γ-tocopherol on days 2-5	Nasal histology, morphology of intraepithelial mucosubstances, eosinophilic inflammation, mucin glycoprotein 5AC expression	γ-tocopherol attenuated ozone+OVA increases in intraepithelial mucosubstances, mucosal eosinophils in nasal and paranasal airways, and MUC5AC expression.

Study	Location	Population/animal model	Exposure (O_3 concentration [ppm], duration)	Health endpoint	Results
Chhabra et al. 2010	India	Guinea pigs (Hartley), M	0.12 ppm, 2 h/day, 4 weeks, sensitized with ovalbumin, with or without vitamin supplementation (vitamin C and E)	Airway responsiveness to inhaled histamine (Sgaw, AHR), BALF measures of antioxidant status and oxidative stress	Antioxidant supplementation attenuated O_3-induced increase in AHR and early and late bronchoconstrictive responses after OVA, increased BALF superoxide, increased plasma MDA, decreased red cell SOD activity.
Kodavanti et al. 1995	NC, USA	Guinea pigs (Hartley), F	0, 0.2, 0.4, and 0.8 ppm, 23 h/day, 7 days, dietary deficiency in ascorbate 1 week prior, during, and 1 week post exposure	BALF cellularity, protein, albumin, antioxidants	Ozone (0.8 ppm) increased BALF protein and albumin and ascorbate deficiency did not change the response. Ascorbate deficiency increased the O_3-induced BALF cells and neutrophils (0.8 ppm). Ozone increased lung and BALF ascorbate, glutathione, and uric acid. No change in lung α-tocopherol.
Valacchi et al. 2009	CA, USA	Mice (SKH-1 hairless mice)	0.8 ppm, 6 h/day, 7 days, supplemented diet with β-carotene for 1 month prior	Proinflammatory markers expression (TNFα, MIP2, HO-1, iNOS) in skin tissue	O_3 increased mRNA expression of TNFα, MIP2, iNOS and HO-1 protein. β-carotene supplementation protected against these increases.
Paquette et al. 1996	MD, USA	Mice (C57BL/6J and C3H/HeJ), M/F	0.3 ppm, 48 and 72 h; pregnant dams fed vitamin A sufficient or deficient diets, litters fed same diet until 6-8 weeks old, then O_3 exposure; group of vitamin A deficient mice were supplemented with vitamin A for 5 days prior to O_3 exposure	BALF cellularity and protein	Increased BALF protein and epithelial cells after O_3 exposure in vitA deficient mice compared to sufficient mice in both strains. O_3-induced PMN and macrophage infiltration was increased in vitA deficient C3 mice compared to sufficient, not in B6.

References

Alexeeff SE, Litonjua AA, Suh H, Sparrow D, Vokonas PS, Schwartz J. 2007. Ozone exposure and lung function: Effect modified by obesity and airways hyperresponsiveness in the va normative aging study. Chest 132:1890-1897.

Alexis N, Urch B, Tarlo S, Corey P, Pengelly D, O'Byrne P, et al. 2000. Cyclooxygenase metabolites play a different role in ozone-induced pulmonary function decline in asthmatics compared to normals. Inhal Toxicol 12:1205-1224.

Arbex AM, de Souza Conceicao GM, Perez Cendon S, Arbex FF, Lopes AC, Providello Moyses E, et al. 2009. Urban air pollution and copd-related emergency room visits. J Epidemiol Community Health 966:777-783.

Auten RL, Potts EN, Mason SN, Fischer B, Huang Y, Foster WM. 2009. Maternal exposure to particulate matter increases postnatal ozone-induced airway hyperreactivity in juvenile mice. Am J Respir Crit Care Med 180:1218-1226.

Barraza-Villarreal A, Sunyer J, Hernandez-Cadena L, Escamilla-Nunez MC, Sienra-Monge JJ, Ramirez-Aguilar M, et al. 2008. Air pollution, airway inflammation, and lung function in a cohort study of mexico city schoolchildren. Environ Health Perspect 116:832-838.

Basha MA, Gross KB, Gwizdala CJ, Haidar AH, Popovich J, Jr. 1994. Bronchoalveolar lavage neutrophilia in asthmatic and healthy volunteers after controlled exposure to ozone and filtered purified air. Chest 106:1757-1765.

Bell ML, Dominici F. 2008. Effect modification by community characteristics on the short-term effects of ozone exposure and mortality in 98 us communities. Am J Epidemiol 167:986-997.

Bennett WD, Hazucha MJ, Folinsbee LJ, Bromberg PA, Kissling GE, London SJ. 2007. Acute pulmonary function response to ozone in young adults as a function of body mass index. Inhal Toxicol 19:1147-1154.

Berhane K, Zhang Y, Linn WS, Rappaport EB, Bastain TM, Salam MT, et al. 2011. The effect of ambient air pollution on exhaled nitric oxide in the children's health study. Eur Respir J 37:1029-1036.

Bils RF. 1970. Ultrastructural alterations of alveolar tissue of mice: Iii. Ozone. Arch Environ Health 20:468-480.

Bosson J, Stenfors N, Bucht A, Helleday R, Pourazar J, Holgate ST, et al. 2003. Ozone-induced bronchial epithelial cytokine expression differs between healthy and asthmatic subjects. Clin Exp Allergy 33:777-782.

Buadong D, Jinsart W, Funatagawa I, Karita K, Yano E. 2009. Association between pm10 and o3 levels and hospital visits for cardiovascular diseases in bangkok, thailand. J Epidemiol 19:182-188.

Burra TA, Moineddin R, Agha MM, Glazier RH. 2009. Social disadvantage, air pollution, and asthma physician visits in toronto, canada. Environ Res 109:567-574.

Bush ML, Asplund PT, Miles KA, Ben-Jebria A, Ultman JS. 1996. Longitudinal distribution of o3 absorption in the lung: Gender differences and intersubject variability. J Appl Physiol 81:1651-1657.

Cakmak S, Dales RE, Judek S. 2006a. Do gender, education, and income modify the effect of air pollution gases on cardiac disease? J Occup Environ Med 48:89-94.

Cakmak S, Dales RE, Judek S. 2006b. Respiratory health effects of air pollution gases: Modification by education and income. Arch Environ Occup Health 61:5-10.

Cakmak S, Dales RE, Vidal CB. 2007. Air pollution and mortality in chile: Susceptibility among the elderly. Environ Health Perspect 115:524-527.

Cakmak S, Dales RE, Angelica Rubio M, Blanco Vidal C. 2011. The risk of dying on days of higher air pollution among the socially disadvantaged elderly. Environ Res 111:388-393.

Carbajal-Arroyo L, Miranda-Soberanis V, Medina-Ramón M, Rojas-Bracho L, Tzintzun G, Solís-Gutiérrez P, et al. 2011. Effect of pm10 and o3 on infant mortality among residents in the mexico city metropolitan area: A case-crossover analysis, 19972005. J Epidemiol Community Health 65:715-721.

Carey SA, Minard KR, Trease LL, Wagner JG, Garcia GJ, Ballinger CA, et al. 2007. Three-dimensional mapping of ozone-induced injury in the nasal airways of monkeys using magnetic resonance imaging and morphometric techniques. Toxicol Pathol 35:27-40.

CDC. 2004. The health consequences of smoking: A report of the Surgeon General. Washington, DC. http://www.cdc.gov/tobacco/data_statistics/sgr/2004/complete_report/index.htm

Chhabra SK, Yasir A, Chaudhry K, Shah B. 2010. Effect of ozone on response to ovalbumin & its modulation by vitamins c & e in sensitized guinea pigs. Indian J Med Res 132:87-93.

Escamilla-Nuñez MC, Barraza-Villarreal A, Hernandez-Cadena L, Moreno-Macias H, Ramirez-Aguilar M, Sienra-Monge JJ, et al. 2008. Traffic-related air pollution and respiratory symptoms among asthmatic children, resident in mexico city: The eva cohort study. Respir Res 9:74.

Fanucchi MV, Plopper CG, Evans MJ, Hyde DM, Van Winkle LS, Gershwin LJ, et al. 2006. Cyclic exposure to ozone alters distal airway development in infant rhesus monkeys. Am J Physiol Lung Cell Mol Physiol 291:L644-L650.

Fortino V, Maioli E, Torricelli C, Davis P, Valacchi G. 2007. Cutaneous mmps are differently modulated by environmental stressors in old and young mice. Toxicol Lett 173:73-79.

Funabashi H, Shima M, Kuwaki T, Hiroshima K, Kuriyama T. 2004. Effects of repeated ozone exposure on pulmonary function and bronchial responsiveness in mice sensitized with ovalbumin. Toxicology 204:75-83.

Halonen JI, Lanki T, Tiittanen P, Niemi JV, Loh M, Pekkanen J. 2009. Ozone and cause-specific cardiorespiratory morbidity and mortality. J Epidemiol Community Health 64:814-820.

Hamade AK, Tankersley CG. 2009. Interstrain variation in cardiac and respiratory adaptation to repeated ozone and particulate matter exposures. Am J Physiol Regul Integr Comp Physiol 296:R1202-R1215.

Hamade AK, Misra V, Rabold R, Tankersley CG. 2010. Age-related changes in cardiac and respiratory adaptation to acute ozone and carbon black exposures: Interstrain variation in mice. Inhal Toxicol 22:84-94.

Hansen CA, Barnett AG, Pritchard G. 2008. The effect of ambient air pollution during early pregnancy on fetal ultrasonic measurements during mid-pregnancy. Environ Health Perspect 116:362-369.

Harkema JR, Plopper CG, Hyde DM, St George JA, Dungworth DL. 1987. Effects of an ambient level of ozone on primate nasal epithelial mucosubstances: Quantitative histochemistry. Am J Pathol 127:90-96.

Hazucha MJ, Folinsbee LJ, Bromberg PA. 2003. Distribution and reproducibility of spirometric response to ozone by gender and age. J Appl Physiol 95:1917-1925.

Henrotin JB, Besancenot JP, Bejot Y, Giroud M. 2007. Short-term effects of ozone air pollution on ischaemic stroke occurrence: A case-crossover analysis from a 10-year population-based study in dijon, france. Occup Environ Med 64:439-445.

Hernandez ML, Lay JC, Harris B, Esther CR, Brickey WJ, Bromberg PA, et al. 2010. Atopic asthmatic subjects but not atopic subjects without asthma have enhanced inflammatory response to ozone. J Allergy Clin Immunol 126:537-544.

HERO (Health and Environmental Research Online). 2014. HERO's scientific studies and other references database. Available: http://hero.epa.gov/

Hill AB. 1965. The environment and disease: Association or causation? Proc R Soc Med 58(5): 295-300.

Horstman DH, Ball BA, Brown J, Gerrity T, Folinsbee LJ. 1995. Comparison of pulmonary responses of asthmatic and nonasthmatic subjects performing light exercise while exposed to a low level of ozone. Toxicol Ind Health 11:369-385.

Hu SC, Ben-Jebria A, Ultman JS. 1994. Longitudinal distribution of ozone absorption in the lung: Effects of respiratory flow. J Appl Physiol 77:574-583.

Joad JP, Kott KS, Bric JM, Peake JL, Plopper CG, Schelegle ES, et al. 2006. Structural and functional localization of airway effects from episodic exposure of infant monkeys to allergen and/or ozone. Toxicol Appl Pharmacol 214:237-243.

Johnston RA, Theman TA, Lu FL, Terry RD, Williams ES, Shore SA. 2008. Diet-induced obesity causes innate airway hyperresponsiveness to methacholine and enhances ozone-induced pulmonary inflammation. J Appl Psychol 104:1727-1735.

Jorres R, Nowak D, Magnussen H, Speckin P, Koschyk S. 1996. The effect of ozone exposure on allergen responsiveness in subjects with asthma or rhinitis. Am J Respir Crit Care Med 153:56-64.

Kan H, London SJ, Chen G, Zhang Y, Song G, Zhao N, et al. 2008. Season, sex, age, and education as modifiers of the effects of outdoor air pollution on daily mortality in shanghai, china: The public health and air pollution in asia (papa) study. Environ Health Perspect 116:1183-1188.

Katsouyanni K, Samet JM, Anderson HR, Atkinson R, Le Tertre A, Medina S, et al. 2009. Air pollution and health: A european and north american approach (aphena). Research Report 142. Boston, MA:Health Effects Institute.

Ko FWS, Tam W, Wong TW, Lai CKW. 2007. Effects of air pollution on asthma hospitalization rates in different age groups in hong kong. Clin Exp Allergy 37:1312-1319.

Kodavanti UP, Costa DL, Dreher KL, Crissman K, Hatch GE. 1995. Ozone-induced tissue injury and changes in antioxidant homeostasis in normal and ascorbate-deficient guinea pigs. Biochem Pharmacol 50:243-251.

Kreit JW, Gross KB, Moore TB, Lorenzen TJ, D'Arcy J, Eschenbacher WL. 1989. Ozone-induced changes in pulmonary function and bronchial responsiveness in asthmatics. J Appl Physiol 66:217-222.

Lee JT, Son JY, Kim H, Kim SY. 2006. Effect of air pollution on asthma-related hospital admissions for children by socioeconomic status associated with area of residence. Arch Environ Occup Health 61:123-130.

Lim Y, Phung AD, Corbacho AM, Aung HH, Maioli E, Reznick AZ, et al. 2006. Modulation of cutaneous wound healing by ozone: Differences between young and aged mice. Toxicol Lett 160:127-134.

Lin M, Stieb DM, Chen Y. 2005. Coarse particulate matter and hospitalization for respiratory infections in children younger than 15 years in toronto: A case-crossover analysis. Pediatrics 116:235-240.

Lin S, Liu X, Le LH, Hwang SA. 2008. Chronic exposure to ambient ozone and asthma hospital admissions among children. Environ Health Perspect 116:1725-1730.

López I, Sánchez I, Bizarro P, Acevedo S, Ustarroz M, Fortoul T. 2008. Ultrastructural alterations during embryonic rats' lung development caused by ozone. J Electron Microsc (Tokyo) 57:19-23.

Mar TF, Koenig JQ. 2009. Relationship between visits to emergency departments for asthma and ozone exposure in greater seattle, washington. Ann Allergy Asthma Immunol 103:474-479.

McDonnell WF, Stewart PW, Smith MV, Pan WK, Pan J. 1999. Ozone-induced respiratory symptoms: Exposure-response models and association with lung function. Eur Respir J 14:845-853.

McDonnell WF, Stewart PW, Smith MV. 2010. Prediction of ozone-induced lung function responses in humans. Inhal Toxicol 22:160-168.

Medina-Ramón M, Schwartz J. 2008. Who is more vulnerable to die from ozone air pollution? Epidemiology 19:672-679.

Middleton N, Yiallouros P, Kleanthous S, Kolokotroni O, Schwartz J, Dockery DW, et al. 2008. A 10-year time-series analysis of respiratory and cardiovascular morbidity in nicosia, cyprus: The effect of short-term changes in air pollution and dust storms. Environ Health 7:39.

Morello-Frosch R, Jesdale BM, Sadd JL, Pastor M. 2010. Ambient air pollution exposure and full-term birth weight in california. Environ Health 9:44.

Mudway IS, Stenfors N, Blomberg A, Helleday R, Dunster C, Marklund SL, et al. 2001. Differences in basal airway antioxidant concentrations are not predictive of individual responsiveness to ozone: A comparison of healthy and mild asthmatic subjects. Free Radic Biol Med 31:962-974.

Nodelman V, Ultman JS. 1999. Longitudinal distribution of chlorine absorption in human airways: A comparison to ozone absorption. J Appl Physiol 87:2073-2080.

Oyarzún M, Dussaubat N, González S. 2005. Effect of 0.25 ppm ozone exposure on pulmonary damage induced by bleomycin. Biol Res 38:353-358.

Paquette NC, Zhang LY, Ellis WA, Scott AL, Kleeberger SR. 1996. Vitamin a deficiency enhances ozone-induced lung injury. Am J Physiol 270:L475-L482.

Paulu C, Smith AE. 2008. Tracking associations between ambient ozone and asthma-related emergency department visits using case-crossover analysis. J Public Health Manag Pract 14:581-591.

Peden DB, Boehlecke B, Horstman D, Devlin R. 1997. Prolonged acute exposure to 0.16 ppm ozone induces eosinophilic airway inflammation in asthmatic subjects with allergies. J Allergy Clin Immunol 100:802-808.

Plopper CG, Smiley-Jewell SM, Miller LA, Fanucchi MV, Evans MJ, Buckpitt AR, et al. 2007. Asthma/allergic airways disease: Does postnatal exposure to environmental toxicants promote airway pathobiology? Toxicol Pathol 35:97-110.

Rivas-Arancibia S, Dorado-Martinez C, Borgonio-Perez G, Hiriart-Urdanivia M, Verdugo-Diaz L, Duran-Vazquez A, et al. 2000. Effects of taurine on ozone-induced memory deficits and lipid peroxidation levels in brains of young, mature, and old rats. Environ Res 82:7-17.

Romieu I, Ramirez-Aguilar M, Moreno-Macias H, Barraza-Villarreal A, Miller P, Hernandez-Cadena L, et al. 2004. Infant mortality and air pollution: Modifying effect by social class. J Occup Environ Hyg 46:1210-1216.

Romieu I, Barraza-Villarreal A, Escamilla-Núñez C, Texcalac-Sangrador JL, Hernandez-Cadena L, Díaz-Sánchez D, et al. 2009. Dietary intake, lung function and airway inflammation in mexico city school children exposed to air pollutants. Respir Res 10:122.

Samet JM, Hatch GE, Horstman D, Steck-Scott S, Arab L, Bromberg PA, et al. 2001. Effect of antioxidant supplementation on ozone-induced lung injury in human subjects. Am J Respir Crit Care Med 164:819-825.

Scannell C, Chen L, Aris RM, Tager I, Christian D, Ferrando R, et al. 1996. Greater ozone-induced inflammatory responses in subjects with asthma. Am J Respir Crit Care Med 154:24-29.

Schelegle ES, Miller LA, Gershwin LJ, Fanucchi MV, Van Winkle LS, Gerriets JE, et al. 2003. Repeated episodes of ozone inhalation amplifies the effects of allergen sensitization and inhalation on airway immune and structural development in rhesus monkeys. Toxicol Appl Pharmacol 191:74-85.

Seal E, Jr, McDonnell WF, House DE. 1996. Effects of age, socioeconomic status, and menstrual cycle on pulmonary response to ozone. Arch Environ Occup Health 51:132-137.

Servais S, Boussouar A, Molnar A, Douki T, Pequignot JM, Favier R. 2005. Age-related sensitivity to lung oxidative stress during ozone exposure. Free Radic Res 39:305-316.

Shore SA, Lang JE, Kasahara DI, Lu FL, Verbout NG, Si H, et al. 2009. Pulmonary responses to subacute ozone exposure in obese vs. Lean mice. J Appl Physiol 107:1445-1452.

Sienra-Monge JJ, Ramirez-Aguilar M, Moreno-Macias H, Reyes-Ruiz NI, Del Rio-Navarro BE, Ruiz-Navarro MX, et al. 2004. Antioxidant supplementation and nasal inflammatory responses among young asthmatics exposed to high levels of ozone. Clin Exp Immunol 138:317-322.

Silverman RA, Ito K. 2010. Age-related association of fine particles and ozone with severe acute asthma in new york city. J Allergy Clin Immunol 125:367-373.

Stafoggia M, Forastiere F, Faustini A, Biggeri A, Bisanti L, Cadum E, et al. 2010. Susceptibility factors to ozone-related mortality: A population-based case-crossover analysis. Am J Respir Crit Care Med 182:376-384.

Tankersley CG, Peng RD, Bedga D, Gabrielson K, Champion HC. 2010. Variation in echocardiographic and cardiac hemodynamic effects of pm and ozone inhalation exposure in strains related to nppa and npr1 gene knock-out mice. Inhal Toxicol 22:695-707.

Thaller EI, Petronella SA, Hochman D, Howard S, Chhikara RS, Brooks EG. 2008. Moderate increases in ambient pm2.5 and ozone are associated with lung function decreases in beach lifeguards. J Occup Environ Med 50:202-211.

Tovalin H, Valverde M, Morandi MT, Blanco S, Whitehead L, Rojas E. 2006. DNA damage in outdoor workers occupationally exposed to environmental air pollutants. Occup Environ Med 63:230-236.

Trenga CA, Koenig JQ, Williams PV. 2001. Dietary antioxidants and ozone-induced bronchial hyperresponsiveness in adults with asthma. Arch Environ Occup Health 56:242-249.

U.S. EPA. 2005. Guidelines for carcinogen risk assessment. EPA/630/P-03/001F. Washington, DC. http://www.epa.gov/cancerguidelines/

U.S. EPA. 2008. Notice of Workshop and Call for Information on Integrated Science Assessment for Ozone. FRL-8722-2; Docket ID EPA-HQ-OAR-2008-0699. Washington, DC.http://www.gpo.gov/fdsys/pkg/FR-2008-09-29/html/E8-22799.htm

U.S. EPA. 2009. Integrated science assessment for particulate matter. EPA/600/R-08/139F. Research Triangle Park, NC. http://cfpub.epa.gov/ncea/cfm/recordisplay.cfm?deid=216546

U.S. EPA. 2011. National Ambient Air Quality Standards. Available: http://www.epa.gov/air/criteria.html U.S. EPA. 2013. Integrated science assessment for ozone and related photochemical oxidants. EPA/600/R-10/076F. Research Triangle Park, NC. http://cfpub.epa.gov/ncea/isa/recordisplay.cfm?deid=247492

U.S. EPA. 2014. EPA Clean Air Scientific Advisory Committee. Available: www.epa.gov/casac

Valacchi G, Pecorelli A, Mencarelli M, Maioli E, Davis PA. 2009. Beta-carotene prevents ozone-induced proinflammatory markers in murine skin. Toxicol Ind Health 25:241-247.

Vancza EM, Galdanes K, Gunnison A, Hatch G, Gordon T. 2009. Age, strain, and gender as factors for increased sensitivity of the mouse lung to inhaled ozone. Toxicol Sci 107:535-543.

Villeneuve PJ, Chen L, Stieb D, Rowe BH. 2006. Associations between outdoor air pollution and emergency department visits for stroke in edmonton, canada. Eur J Epidemiol 21:689-700.

Wagner JG, Jiang Q, Harkema JR, Illek B, Patel DD, Ames BN, et al. 2007. Ozone enhancement of lower airway allergic inflammation is prevented by gamma-tocopherol. Free Radic Biol Med 43:1176-1188.

Wagner JG, Harkema JR, Jiang Q, Illek B, Ames BN, Peden DB. 2009. Gamma-tocopherol attenuates ozone-induced exacerbation of allergic rhinosinusitis in rats. Toxicol Pathol 37:481-491.

Wong CM, Ou CQ, Chan KP, Chau YK, Thach TQ, Yang L, et al. 2008. The effects of air pollution on mortality in socially deprived urban areas in hong kong, china. Environ Health Perspect 116:1189-1194.

Wong CM, Yang L, Thach TQ, Chau PY, Chan KP, Thomas GN, et al. 2009. Modification by influenza on health effects of air pollution in hong kong. Environ Health Perspect 117:248-253.

Zanobetti A, Schwartz J. 2008. Is there adaptation in the ozone mortality relationship: A multi-city case-crossover analysis. Environ Health 7:22.
